# Fluoride binding to an organoboron wire controls photoinduced electron transfer†
†Electronic supplementary information (ESI) available: Synthetic protocols and product characterization data, additional optical spectroscopic, NMR, and cyclic voltammetry data. See DOI: 10.1039/c5sc00964b


**DOI:** 10.1039/c5sc00964b

**Published:** 2015-05-01

**Authors:** Jing Chen, Oliver S. Wenger

**Affiliations:** a Department of Chemistry , University of Basel , St. Johanns-Ring 19 , CH-4056 Basel , Switzerland . Email: oliver.wenger@unibas.ch; b Xiamen Institute of Rare Earth Materials , Chinese Academy of Sciences , Xiamen 361021 , People's Republic of China; c Key Laboratory of Design and Assembly of Functional Nanostructures , Fujian Provincial Key Laboratory of Nanomaterials , Fujian Institute of Research on the Structure of Matter , Chinese Academy of Sciences , People's Republic of China

## Abstract

We demonstrate that the rates for long-range electron transfer can be controlled actively by tight anion binding to a rigid rod-like molecular bridge. Electron transfer from a triarylamine donor to a photoexcited Ru(bpy)_3_^2+^ acceptor (bpy = 2,2′-bipyridine) across a 2,5-diboryl-1,4-phenylene bridge occurs within less than 10 ns in CH_2_Cl_2_ at 22 °C. Fluoride anions bind with high affinity to the organoboron bridge due to strong Lewis base/Lewis acid interactions, and this alters the electronic structure of the bridge drastically. Consequently, a large tunneling barrier is imposed on photoinduced electron transfer from the triarylamine to the Ru(bpy)_3_^2+^ complex and hence this process occurs more than two orders of magnitude more slowly, despite the fact that its driving force is essentially unaffected by fluoride addition. Electron transfer rates in proteins could potentially be regulated *via* a similar fundamental principle, because interactions between charged amino acid side chains and counter-ions can modulate electronic couplings between distant redox partners. In artificial donor-bridge-acceptor compounds, external stimuli have been employed frequently to control electron transfer rates, but the approach of exploiting strong Lewis acid/Lewis base interactions to regulate the tunneling barrier height imposed by a rigid rod-like molecular bridge is conceptually novel and broadly applicable, because it is largely independent of the donor and the acceptor, and because the effect is not based on a change of the driving-force for electron transfer. The principle demonstrated here can potentially be used to switch between conducting and insulating states of molecular wires between electrodes.

## Abstract 2

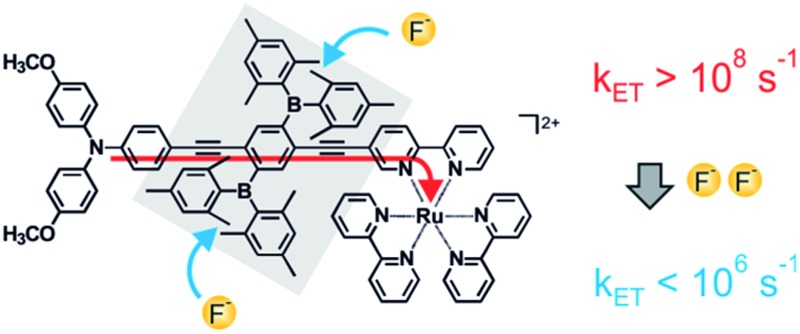
The efficiency of organoboron wires as mediators of long-range electron transfer can be controlled by anion binding.

## 1. Introduction

The electronic structure of an intervening medium between a donor and an acceptor strongly affects the rate at which an electron can be transferred.^1^ Consequently, in artificial donor-bridge-acceptor compounds as well as in biological systems scientists have strived to optimize the electronic structure of molecular bridges in order to accelerate electron transfer over long distances (>10 Å).^2^ Similarly, efforts have been made to optimize electron transport across nanowires between two electrodes.^3^ In several cases it has been possible to modulate the electronic structure of a photoisomerizable molecular bridge or a nanowire with light as an external stimulus to such an extent that electron transfer rates or conductivities could be altered by several orders of magnitude.^4^ In this paper we demonstrate that the rate for long-range electron tunneling across a rigid rod-like organoboron bridge can be controlled by tight fluoride binding to the bridge, without affecting the donor or the acceptor, and without changing the driving-force for electron transfer.

It is well known that fluoride binding alters the electronic structure of organoboron compounds substantially, as this forms the basis for F^–^ or CN^–^ detection in numerous dimesitylboron-substituted molecules and metal complexes.^5^ When using bulky dimesitylboryl groups, the electron-deficient boron atom is well protected from nucleophilic attack, and only small anions such as F^–^ or CN^–^ can bind efficiently. The disruption of p_π_–π* conjugation between the vacant p orbital of boron and the adjacent π-conjugated framework upon anion binding to the boron center causes significant changes in HOMO/LUMO energies.^6^ While organoboron compounds received considerable attention for various optical, electronic, and sensory applications,^7^ they have, to the best of our knowledge, never been used as molecular bridges that mediate electron transfer between covalently attached donors and acceptors, or as well-defined nanowires between two electrodes.

There are many studies in which organoboron units acted as terminal electron acceptors, and upon F^–^ binding electron transfer was suppressed due to a change in driving force.5a,c–e,j,7h,i,k,o,^8^ We investigated a fundamentally different aspect in that we explored how fluoride binding to an organoboron bridge influences electron transfer between distant donors and acceptors which themselves are unaffected by F^–^ addition, *i.e.*, without changing the driving force. In several other prior studies the addition of ions lead to an effect on the luminescence or absorption properties of an oligomer or polymer material, but the fundamental electron transfer properties across the oligomer/polymer material were not investigated.5n,6a,7s,t,^9^

**Scheme 1:**
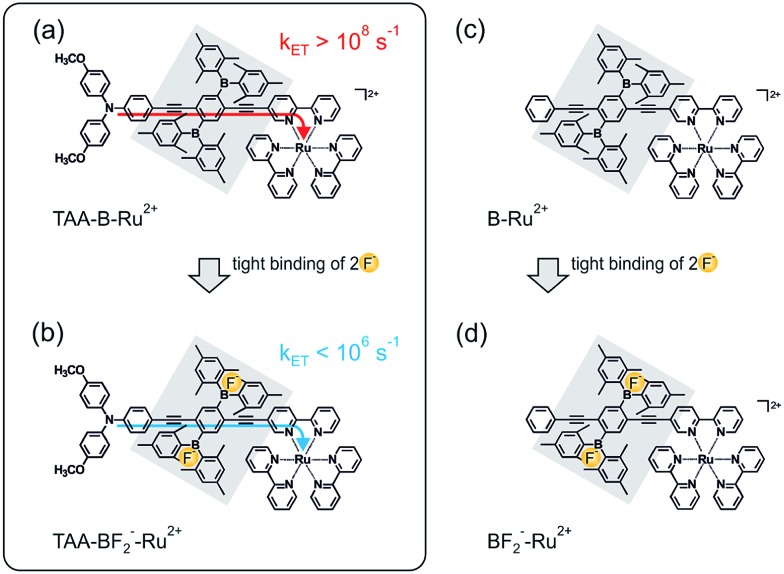
Chemical structure of the **TAA-B-Ru^2+^** dyad (a) and the **B–Ru^2+^** reference compound (c) investigated here, along with an illustration of the key finding from this study: intramolecular photoinduced electron transfer is rapid in **TAA-B-Ru^2+^** in CH_2_Cl_2_ (a), but upon fluoride addition the **TAA-BF_2_^–^-Ru^2+^** species is formed, and the rate constant for intramolecular electron transfer (*k*_ET_) decreases by more than 2 orders of magnitude (b). All compounds in scheme were isolated as PF_6_^–^ salts, see ESI.†

The key compound of this study (**TAA-B-Ru^2+^**, Scheme 1a) is comprised of a triarylamine donor, a 2,5-diboryl-1,4-phenylene bridge (grey shaded unit), and a Ru(bpy)_3_^2+^ acceptor (bpy = 2,2′-bipyridine). Electron transfer from the triarylamine to the photoexcited Ru(bpy)_3_^2+^ complex occurs with a rate constant (*k*_ET_) exceeding 10^8^ s^–1^ in CH_2_Cl_2_ at 22 °C (Scheme 1a). When fluoride anions are bound to the organoboron bridge, the same electron transfer process occurs with a rate constant lower than 10^6^ s^–1^ under identical conditions (Scheme 1b). A reference compound comprised of only the organoboron bridge and the Ru(bpy)_3_^2+^ complex but lacking the triarylamine donor (**B-Ru^2+^**, Scheme 1c) was also investigated.

Many prior studies have used chemical stimuli to control electron transfer rates, for example protons which are bound by amine donors,^10^ cations which are coordinated by aza crown ether donors,^11^ or Lewis acids which interact with quinone acceptors,^12^ just to name a few. However, in the vast majority of cases the chemical stimulus interacts directly with the donor or the acceptor, and control is achieved by changing the driving force for electron transfer. In a few cases alkali cations or ammonium ions were coordinated to molecular bridges bearing crown ether functions.^13^ However, cation binding in these cases is typically rather weak (with association constants on the order of 10^2^–10^5^ M^–1^), and the effects on electron transfer between the covalently attached donors and acceptor were comparatively small. Similarly, anion binding by molecular squares, calixarenes, thioureas, anion-π interactions, *etc.*, often suffers from relatively low binding constants, and in most cases control of electron transfer is achieved by changing its driving-force.^14^ Many of the abovementioned anion-binding systems are structurally rather complex or flexible, but for a future molecular electronics technology anion-responsive wires made from rigid rod-like molecular units are highly desirable.

Our study introduces the concept of anion-controllable rigid rod-like molecular bridges and nanowires which should be broadly applicable because it is largely independent of what donors and acceptors are used and at what driving force the electron transfer operates. In some proteins, electron transfer rates are potentially regulated by a similar fundamental principle because the interaction of ions with charged amino acid side chains can modulate electronic couplings between distant redox partners.^15^

## 2. Results and discussion

### Synthesis

**Scheme 2:**
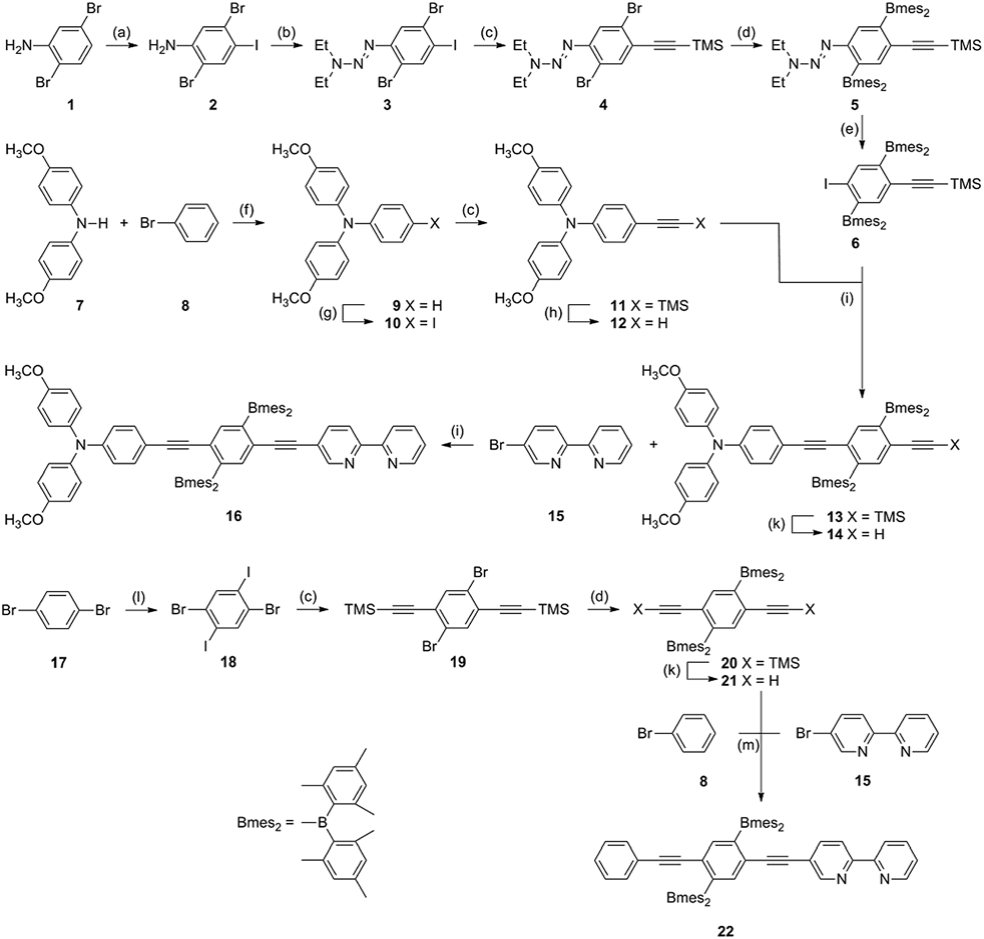
Synthesis of the key ligands. (a) NIS, DMSO; (b) BF_3_·Et_2_O, ^*t*^BuONO, CH_2_Cl_2_, Et_2_NH; (c) TMS–C≡C–H, CuI, Pd(PPh_3_)_2_Cl_2_, Et_3_N, THF; (d) *n*-BuLi, Bmes_2_F, Et_2_O; (e) MeI; (f) P(^*t*^Bu)_3_H^+^BF_4_^–^, Pd(dba)_2_, ^*t*^BuOK, toluene; (g) NIS, DMF; (h) KOH, MeOH, CH_2_Cl_2_; (i) Pd(PPh_3_)_4_, CuI, THF, ^*i*^Pr_2_NH; (k) NaH, THF; (l) I_2_, conc. H_2_SO_4_; (m) Pd(PPh_3_)_4_, CuI, Et_3_N.

The synthesis of the **TAA-B-Ru^2+^** and **B-Ru^2+^** compounds from Scheme 1 involved 15 and 8 steps, respectively, as illustrated by Scheme 2. The key building block for the molecular bridge is the new compound **6** which in principle could be used for the modular (step-by-step) synthesis of mono-disperse oligomers of 2,5-diboryl-1,4-phenylene ethynylene following previously published synthetic protocols for other oligo-*p*-phenylene ethynylene (OPE) wires.^16^ For the synthesis of the **B-Ru^2+^** reference compound, the known symmetrical building block **20** was used instead of compound **6**.7*s* Complete synthetic details and product characterization data are given in the ESI.†

### Fluoride binding to the organoboron unit

**Fig. 1:**
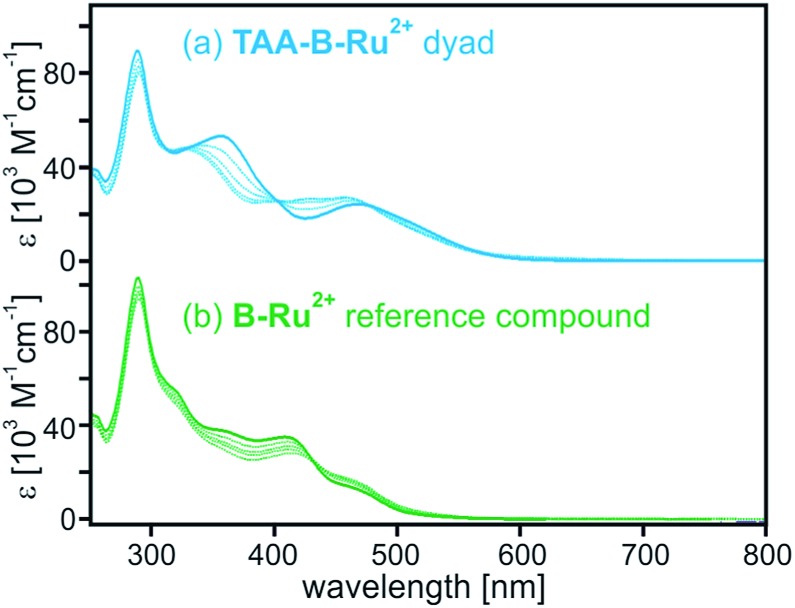
Optical absorption spectra of (a) **TAA-B-Ru^2+^** and (b) **B-Ru^2+^** in CH_2_Cl_2_ at 22 °C. Solid lines: in absence of TBAF; dashed lines: in presence of 1, 2, 3, and 4 equivalents of TBAF.

The solid blue line in Fig. 1a is the optical absorption spectrum of **TAA-B-Ru^2+^** in CH_2_Cl_2_ at 22 °C. The band maximizing at 468 nm is caused by MLCT transitions of the Ru(bpy)_3_^2+^ unit, and the band at 290 nm is due to bpy-localized π–π* transitions. The absorption band centered at 357 nm is absent in the UV-Vis spectrum of Ru(bpy)_3_^2+^ and is attributed to charge transfer from the triarylamino-group to the dimesitylboron-decorated bridging unit. The spectrum of reference compound **B-Ru^2+^** in Fig. 1b (solid green line) exhibits the same MLCT and bpy-localized π–π* bands, but the N → B charge transfer band around 357 nm is missing because ligand **22** lacks the triarylamino-group.

For both compounds spectral changes are easily detectable upon addition of 1–4 equivalents of TBAF (tetra-*n*-butylammonium fluoride) (dashed lines in Fig. 1). The spectral changes observed for **TAA-B-Ru^2+^** are somewhat more dramatic than those observed for **B-Ru^2+^** because the abovementioned N → B charge transfer band around 357 nm disappears upon fluoride binding to **TAA-B-Ru^2+^**. This is a well-known phenomenon because it forms the basis for fluoride detection in many sensor materials.5a–n,^6^,^7^

In the ESI† we report titration curves displaying the absorbance of CH_2_Cl_2_ solutions with known **TAA-B-Ru^2+^** (Fig. S1†) and **B-Ru^2+^** concentrations (Fig. S2†) at selected wavelengths as a function F^–^ concentration. The two data sets were analyzed in terms of a 1 : 2 binding model using the Specfit software, *i.e.*, it was assumed that two fluoride anions bind per **TAA-B-Ru^2+^** or **B-Ru^2+^** molecule because they both contain two dimesitylboron units. The obtained fits to the experimental titration curves are reasonably good (Fig. S1 and S2†), unlike what is obtained with a simpler 1 : 1 binding model. So-called component spectra used to obtain the fits with the 1 : 2 binding model are included in the ESI (Fig. S3 and S4†). The cumulative binding constants for the formation of 1 : 1 (β_1,1_) and 1 : 2 (β_1,2_) adducts obtained in this manner are summarized in Table 1. In both **TAA-B-Ru^2+^** and **B-Ru^2+^** the first fluoride anion binds with an association constant (*K*_A_) on the order of 10^7^ M^–1^ while the second F^–^ binds with *K*_A_ = 10^5^–10^6^ M^–1^. These values are in line with fluoride binding constants reported earlier for related organoboron compounds in similarly apolar solution.5f,7b,o,8b,e,^17^

**Table 1:** Cumulative fluoride binding constants^*a*^

	log(β_1,1_)	log(β_1,2_)
**TAA-B-Ru^2+^**	6.9 ± 0.4	11.9 ± 0.4
**B-Ru^2+^**	7.0 ± 0.7	13.0 ± 0.8

^*a*^In dry CH_2_Cl_2_ at 22 °C. Determined on the basis of the UV-Vis absorption data from Fig. 1 and S1–S4 using commercial 1.0 M TBAF solution in THF.

Addition of TBAF to CD_2_Cl_2_ solutions of **TAA-B-Ru^2+^** and **B-Ru^2+^** leads to the appearance of a resonance at –170 ppm in the ^19^F NMR spectrum (Fig. S5 and S6†), and when excess TBAF is present an additional resonance appears at –128 ppm. The former is characteristic for dimesitylboron-bound fluoride, the latter is due to free F^–^.7b,8b Our ^19^F NMR experiment is unable to distinguish between the two chemically slightly distinct fluoride binding sites present in both **TAA-B-Ru^2+^** and **B-Ru^2+^**, but this is not uncommon.7b,8b With ^11^B NMR spectroscopy one observes the appearance of a resonance at 5 ppm and the disappearance of a resonance at 80 ppm upon fluoride addition (Fig. S7 and S8†), both indicative of F^–^ binding to dimesitylboron.7b,8b,17c Thus it is clear that F^–^ binding occurs at the B atoms, compatible with other recently explored organoboron systems containing Ru(bpy)_3_^2+^ (or similar) complex moieties.5d,5e,17b,^18^ Furthermore it is clear that TBAF solution usually contains some residual water, and given the high hydration enthalpy of F^–^ this is undesirable, and it will lead to lower (apparent) binding constants. However, use of TBAF solution is common practice,5a–n,^7^ and we can exclude that any of the effects reported in the following arise just from the addition of water. The hexafluorophosphate counter-anions of **TAA-B-Ru^2+^** and **B-Ru^2+^** are sterically too demanding in order to interact strongly with our organoboron units,5a–n,^7^ and hence even the use of 0.1 M TBAPF_6_ as a supporting electrolyte for cyclic voltammetry (see below) is unproblematic.17a,b,^19^

### Photoinduced electron transfer and electrochemistry in absence of fluoride

Compared to Ru(bpy)_3_^2+^ or the **B-Ru^2+^** reference compound the **TAA-B-Ru^2+^** dyad is essentially non-luminescent in de-oxygenated CH_2_Cl_2_ at 22 °C (Fig. S9a†). The lowest-energetic ^3^MLCT excited state of the **B–Ru^2+^** reference compound has a lifetime (*τ*) of 2040 ns under these conditions (Table 2), but in the **TAA-B-Ru^2+^** dyad the respective excited state depopulates within less than 10 ns (Fig. S9b†). Evidently, the ^3^MLCT excited state of the **TAA-B-Ru^2+^** dyad is quenched by an efficient nonradiative process.

**Table 2:** Lifetimes of excited states in de-oxygenated CH_2_Cl_2_ at 22 °C

	^3^MLCT^*a*^ [ns]	^3^IL^*b*^ [ns]
Ru(bpy)_3_^2+^	650	
**B-Ru^2+^**	2040	
**BF_2_^–^-Ru^2+^**	1120	7160
**TAA-B-Ru^2+^**	<10	
**TAA-BF_2_^–^-Ru^2+^**	380	7350

^*a*^Emissive state, measured by time-resolved luminescence and transient absorption.

^*b*^Dark state, measured by transient absorption. Lifetimes extracted from the data in Fig. 6, S9b, S10, S12 and S14. Excitation occurred at 532 nm with laser pulses of ∼10 ns duration. Detection was at 620 nm for luminescence and at the wavelengths reported in the text or in the ESI for transient absorption. For more detailed discussion of these lifetimes see the ESI. All lifetime values are associated with an experimental uncertainty of ±5%.

**Fig. 2:**
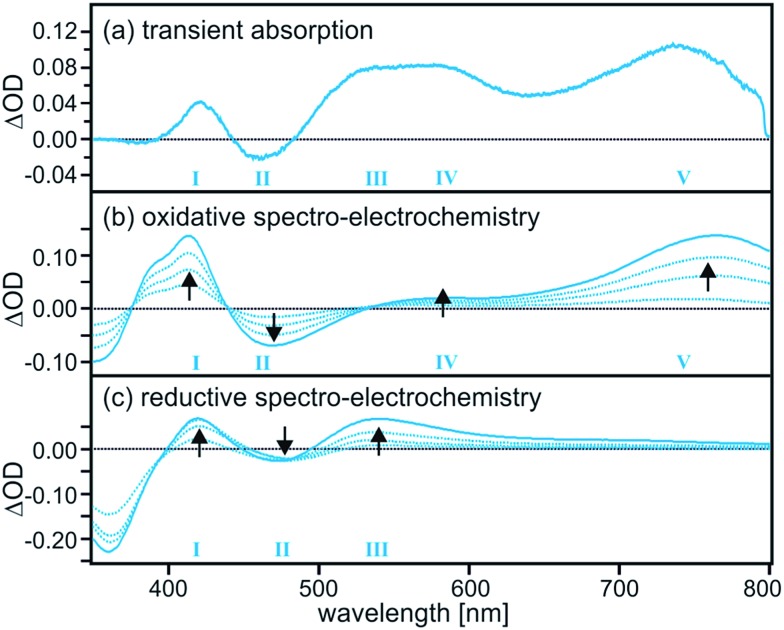
(a) Transient difference spectrum measured after excitation of a 2 × 10^–5^ M solution of **TAA-B-Ru^2+^** in de-oxygenated CH_2_Cl_2_ at 532 nm with laser pulses of ∼10 ns duration (no fluoride present). The spectrum was time-integrated over the first 200 ns following the laser pulses. (b) Series of UV-Vis difference spectra obtained after different time intervals while applying a potential of 0.2 V *vs.* Fc^+^/Fc to a solution of **TAA-B-Ru^2+^** in CH_2_Cl_2_. (c) Analogous series of spectra obtained from the same solution while applying a potential of –1.9 V *vs.* Fc^+^/Fc. In (b) and (c) the UV-Vis spectrum recorded before applying any potential served as a baseline. See text for additional details.

Based on the transient absorption and spectro-electrochemical data in Fig. 2 the efficient nonradiative excited-state quenching process in **TAA-B-Ru^2+^** can be identified unambiguously as intramolecular electron transfer from the triarylamine unit to the photoexcited Ru(bpy)_3_^2+^ moiety. The transient difference spectrum in Fig. 2a was recorded after excitation of 2 × 10^–5^ M **TAA-B-Ru^2+^** in de-oxygenated CH_2_Cl_2_ at 22 °C using laser pulses of 532 nm wavelength and ∼10 ns duration. Selective excitation into the MLCT absorption band was followed by time-integration of the resulting transient difference spectrum over an interval of 200 ns using a iCCD camera. One observes a weak band at 430 nm, a bleach at 465 nm, a broad spectral feature which seems to be due to superposed bands with local maxima near 540 and 580 nm, and finally a well-separated band peaking at 740 nm. These five spectral features (numbered *I*–*V* in Fig. 2a) clearly indicate the formation of oxidized triarylamine and reduced ruthenium complex as will be demonstrated in the following.

The series of spectro-electrochemical data in Fig. 2b and c are difference spectra in which the UV-Vis spectrum of **TAA-B-Ru^2+^** in CH_2_Cl_2_ (prior to applying any potential) was used as a baseline. For measurement of the spectra in Fig. 2b an electrochemical potential of 0.2 V *vs.* Fc^+^/Fc was applied with a Pt grid electrode. This is sufficient for oxidation of the triarylamine unit of **TAA-B-Ru^2+^** (see below), but neither the Ru(bpy)_3_^2+^ moiety nor the bridging organoboron unit will undergo any oxidation at this potential. Thus, the data in Fig. 2b show the spectral changes associated with oxidation of triarylamine to its monocationic form (oxidation to the dication requires more positive potentials, see below).^20^ The longer the potential is applied, the more readily detectable the spectral changes become (black arrows). For the spectra in Fig. 2c an electrochemical potential of –1.9 V *vs.* Fc^+^/Fc was applied, and this lead to one-electron reduction of the Ru(bpy)_3_^2+^ moiety of **TAA-B-Ru^2+^**. Neither the triarylamine unit nor the organoboron bridge can be reduced at this potential (see below). Comparison of Fig. 2a with 2b and c clearly shows that all spectral features observed in the transient absorption spectrum can be explained by combined triarylamine oxidation and Ru(bpy)_3_^2+^ reduction. This is conclusive evidence for intramolecular photoinduced electron transfer and the formation of a photoproduct that we abbreviate as **TAA^+^-B-Ru^+^** in the following.

**Fig. 3:**
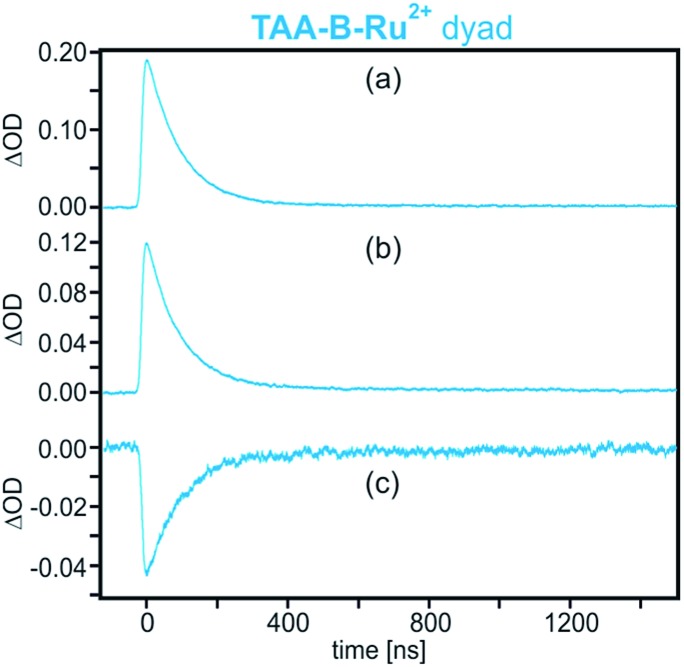
Decays of the transient absorption signals measured on a 2 × 10^–5^ M solution of **TAA-B-Ru^2+^** in de-oxygenated CH_2_Cl_2_ after excitation at 532 nm with laser pulses of ∼10 ns duration. Detection occurred at (a) 740 nm (band V in Fig. 2a), (b) 580 nm (band IV in Fig. 2a), and (c) 460 nm (band II in Fig. 2a).

The transient absorption signals at 740 and 580 nm including the bleach at 460 nm (bands V, IV, and II in Fig. 2a) all exhibit the same temporal evolution (Fig. 3). Each signal reaches its maximum intensity immediately after the laser pulse and decays with a time constant of 95 ns in de-oxygenated CH_2_Cl_2_ at 22 °C. This indicates that photoinduced electron transfer occurs within ≤10 ns (rate constant *k*_ET_ ≥ 10^8^ s^–1^ in Scheme 1a), and the reverse (thermal) electron transfer to reinstate the starting material in the electronic ground state takes place with a time constant of 95 ns (*k*_bET_ = 1.05 × 10^7^ s^–1^).

**Fig. 4:**
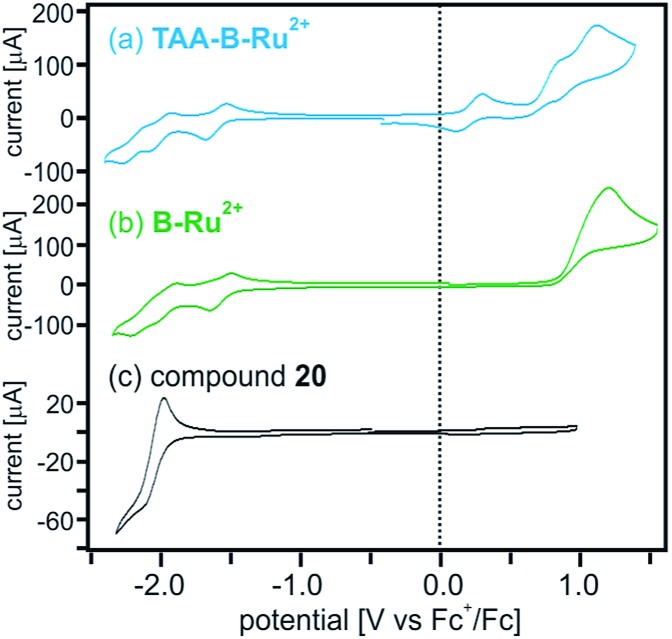
Cyclic voltammograms of (a) **TAA-B-Ru^2+^**, (b) **B-Ru^2+^**, and (c) bridge reference molecule **20** (molecular structure shown in Scheme 2) in CH_2_Cl_2_. The supporting electrolyte was 0.1 M TBAPF_6_. The potential scan rate was 0.1 V s^–1^ in all cases. The shoulder wave at 0.85 V in (a) is due to oxidation of TAA^+^ to TAA^2+^.20*a* All other redox potentials extracted from this data set are summarized in Table 3.

The reaction free energies for photoinduced electron transfer (Δ*G*0ET) and for reverse (thermal) electron transfer (Δ*G*0bET) can be estimated on the basis of electrochemical potentials determined by cyclic voltammetry in dry CH_2_Cl_2_ in presence of 0.1 M TBAPF_6_. In the voltammogram of **TAA-B-Ru^2+^** (Fig. 4a) oxidation of the triarylamine to its monocationic form manifests as a reversible wave at 0.2 V *vs.* Fc^+^/Fc.20a,c–e Oxidation to the triarylamine dication is seen as a quasi-reversible wave at 0.8 V,20*a* superposed on the (irreversible) oxidation of Ru(ii) to Ru(iii) with a peak potential of 1.1 V *vs.* Fc^+^/Fc. On the reductive side, the first (reversible) wave at –1.6 V is due to reduction of a bpy-ligand.20e,^21^ Comparison with the voltammogram of the **B-Ru^2+^** reference compound (Fig. 4b) and with the voltammogram of organoboron bridging unit **20** (Fig. 4c; see Scheme 2 for chemical structure) supports our assignments of redox waves to the individual molecular components of **TAA-B-Ru^2+^**. The voltammogram of compound **20** shows that reduction of the organoboron bridging unit occurs at –2.1 V *vs.* Fc^+^/Fc, in line with previously reported reduction potentials for related species.5h,7k,8e,19b,^22^ All redox potentials are summarized in Table 3.

**Table 3:** Electrochemical potentials in CH_2_Cl_2_ in units of volts *vs.* Fc^+^/Fc^*a*^

	boryl^0/–^	bpy^0/–^	TAA^+/0^	Ru^III/II^
**TAA-B-Ru^2+^**	–2.2	–1.6	0.2	1.1
**B-Ru^2+^**	–2.2	–1.6		1.2
Ru(bpy)_3_^2+^		–1.7		0.9
Compound **20**	–2.1			
**TAA-BF_2_^–^-Ru^2+^**		–1.5	0.2	

^*a*^The electrolyte was 0.1 M TBAPF_6_, the voltage scan rate was 0.1 V s^–1^. Determined from the data in Fig. 4 and S15. The chemical structure of compound **20** is shown in Scheme 2. Ru(bpy)_3_^2+^ denotes the reference complex with three identical (un-substituted) bpy ligands.

Based on the electrochemical potential for oxidation of triarylamine to its monocationic form (0.2 V), the electrochemical potential for one-electron reduction of the Ru(bpy)_3_^2+^ moiety (–1.6 V), the MLCT energy of the Ru(bpy)_3_^2+^ unit (2.1 eV),21*b* and a donor–acceptor distance of ∼15 Å (geometrical triarylamine-N–Ru distance) one can use the Weller equation to estimate a reaction free energy of Δ*G*0ET ≈ –0.3 eV for photoinduced electron transfer in **TAA-B-Ru^2+^** in CH_2_Cl_2_.^23^ Thermal electron transfer in the reverse direction then occurs with Δ*G*0bET ≈ –1.8 eV. As noted above, the photoinduced reaction occurs with *k*_ET_ ≥ 10^8^ s^–1^ whereas for the thermal reverse reaction one finds *k*_bET_ = 1.05 × 10^7^ s^–1^. Thus, the thermal reverse reaction is slower than photoinduced electron transfer despite a substantially higher driving force, indicating that the thermal process occurs in the so-called Marcus inverted region,^24^ as observed before for several other examples of simple donor–acceptor dyads with d^6^ metal diimine photosensitizers.20c–e,^25^ However, the most important result until now is that photoinduced electron transfer from the triarylamine to the Ru(bpy)_3_^2+^ unit of **TAA-B-Ru^2+^** in de-oxygenated CH_2_Cl_2_ at 22 °C occurs with a rate constant of *k*_ET_ ≥ 10^8^ s^–1^ (Scheme 1a).

### Photophysics and electrochemistry in presence of fluoride

When adding 4 equivalents of TBAF to a 2 × 10^–5^ M solution of **TAA-B-Ru^2+^** in dry CH_2_Cl_2_ one reaches conditions under which >99% of all dyads have two F^–^ anions bound to the organoboron bridge, based on the association constants in Table 1. Thus, the resulting solution contains almost exclusively a species that we will refer to as **TAA-BF_2_^–^-Ru^2+^** (Scheme 1b). This is much different from many previously explored systems in which ion binding to, *e.g.* crown ether donors or ureas, is often orders of magnitude weaker.^11^,^13^,^14^ In this regard the tight Lewis acid/Lewis base interaction between the organoboron unit and F^–^ stands out.7b,^26^

**Fig. 5:**
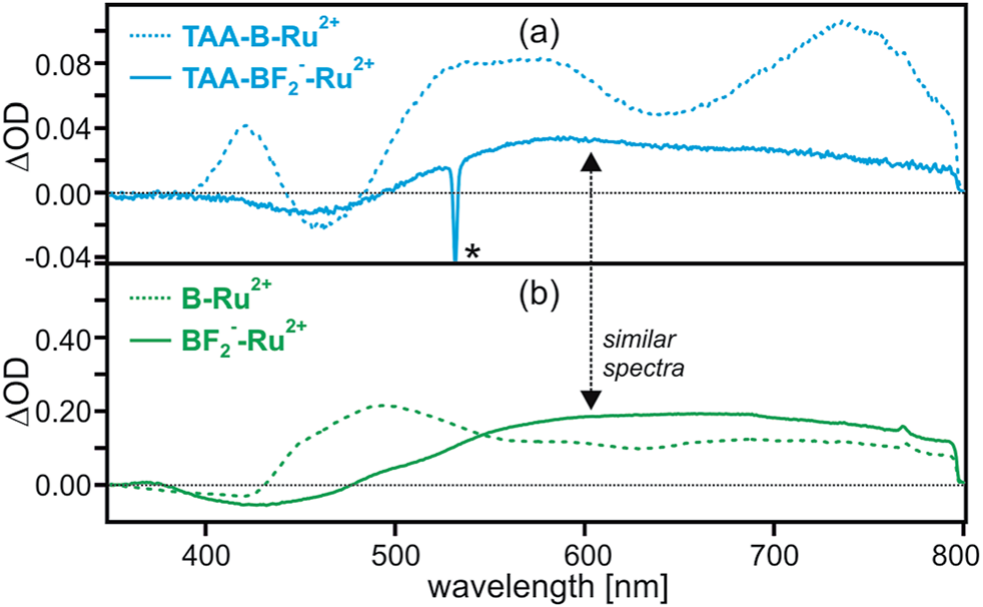
Transient difference spectra measured after excitation of 2 × 10^–5^ M solutions of (a) **TAA-B-Ru^2+^** and (b) **B-Ru^2+^** in de-oxygenated CH_2_Cl_2_ at 532 nm with laser pulses of ∼10 ns duration in absence (dotted traces) and in presence of 4 equivalents of TBAF (solid traces) at 22 °C. All spectra were time-integrated over the first 200 ns following the laser pulses. The individual spectra probe the following species: **TAA-B-Ru^2+^** (dotted blue trace in (a)), **TAA-BF_2_^–^-Ru^2+^** (solid blue trace in (a)), **B-Ru^2+^** (dotted green trace in (b)), and **BF_2_^–^-Ru^2+^** (solid green trace in (b)). The spike marked with an asterisk (*) is due to scattered laser light.

When performing the exact same pump-probe experiment with **TAA-BF_2_^–^-Ru^2+^** as before with **TAA-B-Ru^2+^**, one obtains the transient difference spectrum shown as a solid blue trace in Fig. 5a. For reference, the spectrum of **TAA-B-Ru^2+^** from Fig. 2a is reproduced in Fig. 5a as a dashed trace. Clearly the transient difference spectra of **TAA-BF_2_^–^-Ru^2+^** and **TAA-B-Ru^2+^** are very different. In order to understand the transient difference spectrum of **TAA-BF_2_^–^-Ru^2+^**, an analogous pump-probe experiment with the reference dyad **B-Ru^2+^** (chemical structure shown in Scheme 1c) is most insightful. When adding 4 equivalents of TBAF to a 2 × 10^–5^ M solution of **B-Ru^2+^** in dry CH_2_Cl_2_, two fluoride anions are tightly bound to the organoboron unit in >99% of all **B-Ru^2+^** reference molecules present (Table 1), resulting in a species that we will refer to as **BF_2_^–^-Ru^2+^** in the following. The transient difference spectrum of **BF_2_^–^-Ru^2+^** shown as a solid green trace in Fig. 5b was obtained under the exact same conditions as that of **TAA-BF_2_^–^-Ru^2+^** in Fig. 5a. The solid traces in Fig. 5a and b are virtually identical, indicating that the same photoproduct is formed in the dyad and in the reference compound when F^–^ is bound. Since the reference compound lacks the triarylamine donor, this observation strongly suggests that intramolecular photoinduced electron transfer from the triarylamine to Ru(bpy)_3_^2+^ does no longer occur to a significant extent in **TAA-BF_2_^–^-Ru^2+^**.

**Fig. 6:**
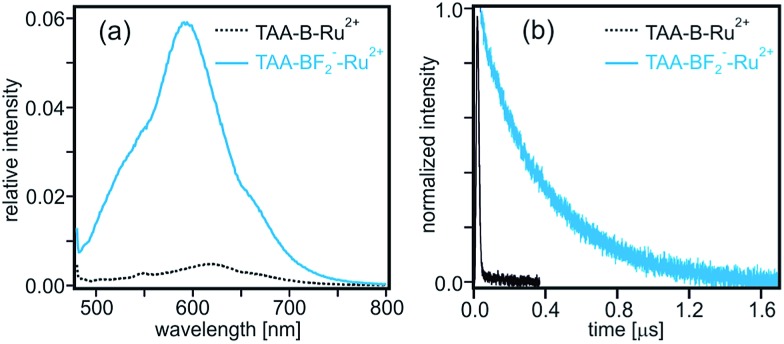
(a) Luminescence of **TAA-B-Ru^2+^** (dotted black trace) and **TAA-BF_2_^–^-Ru^2+^** (solid blue trace) in de-oxygenated CH_2_Cl_2_ after excitation at 470 nm. The relative intensities of the spectra are corrected for differences in absorbance at the excitation wavelength. (b) Luminescence decay of **TAA-B-Ru^2+^** (black trace) and **TAA-BF_2_^–^-Ru^2+^** (blue trace) in de-oxygenated CH_2_Cl_2_ at 22 °C. Excitation occurred at 532 nm, detection was at 620 nm.

Moreover, whereas the **TAA-B-Ru^2+^** dyad without any bound F^–^ ions is essentially non-luminescent in de-oxygenated CH_2_Cl_2_ at 22 °C (*τ* < 10 ns, Table 2, black trace in Fig. 6b), the **TAA-BF_2_^–^-Ru^2+^** compound exhibits ^3^MLCT luminescence with a lifetime (*τ*) of 380 ns under identical conditions (blue traces in Fig. 6 and Table 2). This lifetime corresponds to a ^3^MLCT decay rate constant of *k*_obs_ = 2.6 × 10^6^ s^–1^, which must be equal to the sum of all rate constants for radiative and nonradiative relaxation processes occurring from the ^3^MLCT state. Thus it seems reasonable to conclude that *k*_ET_ < 10^6^ s^–1^ for **TAA-BF_2_^–^-Ru^2+^** (Scheme 1b).

The remaining open question then is: “What exactly *is* the photoproduct observed for **TAA-BF_2_^–^-Ru^2+^** and **BF_2_^–^-Ru^2+^** in Fig. 5?”. The observation of ^3^MLCT luminescence in presence of fluoride strongly suggests that the spectral signature of the ^3^MLCT excited state will contribute substantially to the transient difference spectra shown as solid traces in Fig. 5a and b. This is indeed the case, but the situation is slightly more complicated because there is a contribution from a dark (*i.e.*, non-luminescent) photoproduct in both cases. This conclusion is reached on the basis of the temporal evolution of the transient absorption signals from Fig. 5a and b at various detection wavelengths (Fig. S10†). For **TAA-BF_2_^–^-Ru^2+^** in de-oxygenated CH_2_Cl_2_, the transient absorption signals at 460, 580, and 740 nm all decay in a bi-exponential manner with a fast decay component that corresponds to the ^3^MLCT luminescence lifetime (380 ns) and a slow decay component of 7350 ns (Table 2 and Fig. S10a†). At all three detection wavelengths the intensity ratio of fast *versus* slow decay components is roughly 40%:60%. When performing the exact same experiments with **BF_2_^–^-Ru^2+^**, one also finds bi-exponential decays. The faster decay component (1120 ns, Table 2) again corresponds to the ^3^MLCT luminescence lifetime (Fig. S14d†) whereas the slower decay component (7160 ns, Fig. S10b and S14a–c†) is not observed in luminescence. The relative intensities of fast and slow decay components are again roughly 40% *versus* 60%, at all three detection wavelengths. Thus, the ^3^MLCT state is clearly detectable in transient absorption, and the fact that the resulting spectrum is substantially different from that of the ^3^MLCT state of isolated Ru(bpy)_3_^2+^ is no surprise given the presence of a functionalized bpy ligand in both **TAA-BF_2_^–^-Ru^2+^** and **BF_2_^–^-Ru^2+^**.^27^ The longer-lived dark state is attributed to a triplet state localized on the functionalized bpy ligands (molecules **16** and **22** in Scheme 2).^28^ Further details regarding possible population of this ^3^IL (IL = intraligand) dark state (including additional spectroscopic data for **B-Ru^2+^**) are given only in the ESI† because this issue is of no further interest for the present study.

Clearly the most important finding here is that fluoride binding to **TAA-B-Ru^2+^** in CH_2_Cl_2_ slows down intramolecular electron transfer from the triarylamine to photoexcited Ru(bpy)_3_^2+^ by more than two orders of magnitude, the rate constant *k*_ET_ decreases from >10^8^ s^–1^ to <10^6^ s^–1^.

### Physical origin of the electron transfer rate decrease upon fluoride binding

As noted in the introduction, in the vast majority of prior studies in which a chemical stimulus was used to regulate electron transfer rates, this has occurred by direct interaction of the chemical stimulus with either the donor or the acceptor.^10^–^12^ Consequently, control of electron transfer occurred by modulating its driving force (Δ*G*0ET). In the **TAA-B-Ru^2+^** dyad the chemical stimulus (F^–^) interacts with the bridge, and this is comparatively rare.^13^ Importantly, interaction of F^–^ with the organoboron bridge does not affect the driving force for intramolecular electron transfer from triarlyamine to photoexcited Ru(bpy)_3_^2+^ to a significant extent, because the donor and acceptor redox potentials stay largely unaffected. The cyclic voltammogram of **TAA-BF_2_^–^-Ru^2+^** (Fig. S15†) is less clean than that of **TAA-B-Ru^2+^** (Fig. 4a) due to the presence of fluoride, but one can readily extract electrochemical potentials of 0.2 V *vs.* Fc^+^/Fc for triarylamine oxidation and –1.5 V *vs.* Fc^+^/Fc for one-electron reduction of the Ru(bpy)_3_^2+^ unit (Table 3). Consequently, the driving force for photoinduced electron transfer in **TAA-BF_2_^–^-Ru^2+^** is Δ*G*0ET = –0.4 eV, very similar to what has been found above in absence of F^–^ (Δ*G*0ET = –0.3 eV).

Thus, in principle there is still sufficient driving force for photoinduced electron transfer in **TAA-BF_2_^–^-Ru^2+^**, yet this process is kinetically not competitive with other excited-state deactivation pathways. We attribute this to a decrease in electronic coupling strength (H_DA_) between the triarylamine donor and the Ru(bpy)_3_^2+^ acceptor. According to superexchange theory, H_DA_ depends strongly on the so-called tunneling energy gap,^29^*i.e.*, a quantity which is strongly dependent on the HOMO/LUMO energies of the bridging units mediating long-range electron tunneling.1a,2g,4g,^30^ An alternative and equally valid view (discussed in more detail below) is that the barrier height associated with the tunneling process depends crucially on the redox potentials of the bridging units.1a,2g,^31^ As noted in the introduction, F^–^ binding to organoboron units strongly affects their HOMO/LUMO energies due to disruption of p_π_–π* conjugation between the boron atom and the π-system to which it is attached.7b,f,s Moreover, and perhaps even more importantly, the resulting organofluoroborate species is anionic, and consequently the LUMO is energetically raised compared to the organoboron unit before F^–^ addition. The π-acceptor capacity of the organofluoroborate is substantially lower.

In free **TAA-B-Ru^2+^** without fluoride, photoinduced charge transfer is likely to proceed *via* an *electron* (rather than *hole*) tunneling mechanism for which the LUMO energy of the organoboron bridge is relevant.2j,11c,^32^ In the *electron* tunneling picture, the energy of the (virtual) one-electron reduced state of the organoboron bridge determines the height of the tunneling barrier, hence it is the LUMO energy of the bridge which matters. For *hole* tunneling, the energy of the (virtual) one-electron oxidized state of the organoboron bridge would be relevant, and given the fact that the bridge is very electron-deficient prior to F^–^ binding, this can be expected to lead to a substantially higher barrier than in the case of the *electron* tunneling model.

**Scheme 3:**
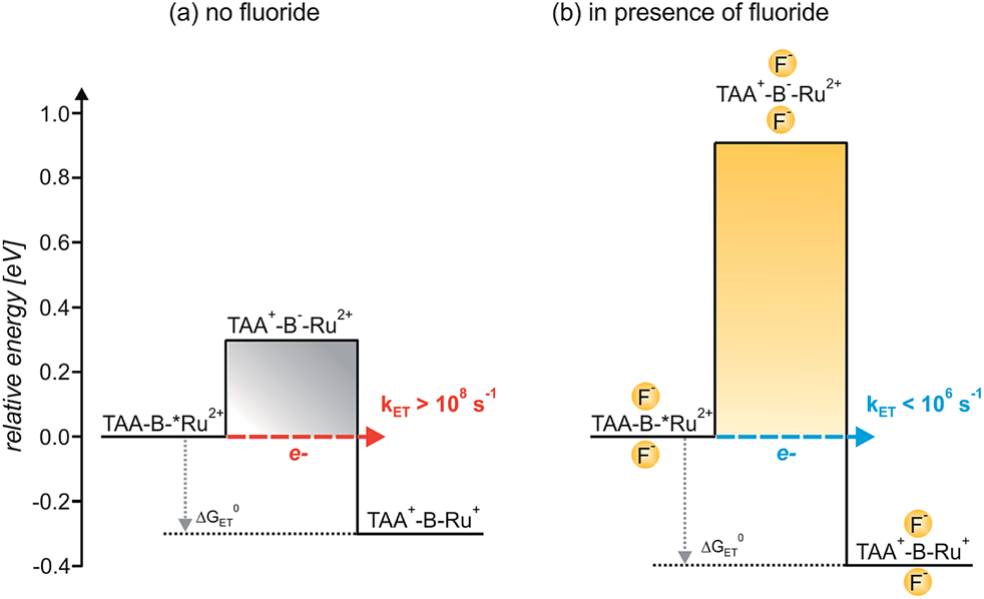
Barrier heights and reaction free energies (Δ*G*0ET) for intramolecular electron transfer from triarylamine to photoexcited Ru(bpy)_3_^2+^ in the **TAA-B^–^-Ru^2+^** dyad in absence (a) and in presence of bound F^–^ anions (b). In presence of 4 eq. of F^–^ (b), **TAA-BF_2_^–^-Ru^2+^** is the predominant species in CH_2_Cl_2_ solution due to tight anion binding, see text. The asterisk (*) denotes the ^3^MLCT-excited Ru(bpy)_3_^2+^ moiety. The reaction free energies (Δ*G*0ET) were estimated on the basis of the electrochemical potentials in Table 3 using the Weller equation as described in the text.^23^ The barrier heights were estimated in analogous manner because they correspond to the reaction free energies for charge injection from the triarylamine donor into the organoboron bridge out of the ^3^MLCT-excited dyad. The reduction potential of the organoboron bridge with 2 attached F^–^ ions was estimated as described in the text.

In the simplistic picture used in Scheme 3a the energy difference between the initial TAA-B-*Ru^2+^ state (the asterisk denotes the ^3^MLCT excited species) and the (virtual) intermediate **TAA^+^-B^–^-Ru^2+^** (comprised of oxidized donor, reduced bridge, and terminal acceptor in its electronic ground state) defines the barrier height for electron tunneling from the triarylamine to the photoexcited ruthenium complex. Based on the electrochemical potentials in Table 3 and the Weller equation, the height of this barrier is ∼0.3 eV. Tunneling across such a barrier can readily occur on the sub-nanosecond timescale.1a,2c,g,i,30a,^33^ When two fluoride anions bind to the organoboron bridge, its reduction potential is expected to shift to significantly more negative values.17a,19a,^34^ We have not been able to measure the respective potential and were unable to find reduction potentials of comparable organofluoroborate species in the literature. However, based on electrochemical studies in chemically related systems it seems reasonable to expect a cathodic shift of at least ∼0.3 V per bound anionic charge,5h,34a,^35^ increasing the barrier height for photoinduced electron tunneling by at least ∼0.6 eV when two fluorides are bound (Scheme 3b). Such an increase in barrier height is expected to decrease the tunneling probability by several orders of magnitude and can explain why photoinduced electron transfer is no longer observed in **TAA-BF_2_^–^-Ru^2+^**.^36^ On the other hand, the electron-rich organofluoroborate bridge is expected to have a significantly lower oxidation potential than its electron-deficient organoboron counterpart without fluorides, and this might result in an increasingly efficient *hole* tunneling pathway.2*j* However, this effect seems to be less important, presumably because the barrier for hole tunneling remains relatively large. More theoretical assessments are certainly possible but beyond the scope of this paper.^37^

## 3. Conclusions

Organoboron compounds have received considerable attention in recent years and many scientists have exploited the change in electronic structure associated with fluoride or cyanide binding to such materials.5a–n,^6^,^7^ Even though the interest of this class of compounds for various optical, electronic, and sensory applications has been pointed out numerous times,^7^ curiously, there have been no fundamental studies of photoinduced electron transfer across organoboron bridges until now.^9^

The key effect observed in this study is illustrated by Scheme 1a and b, and its physical origin is explained graphically by Scheme 3. In the **TAA-B-Ru^2+^** dyad, electron transfer from the triarylamine to the photoexcited Ru(bpy)_3_^2+^ complex across the 2,5-diboryl-1,4-phenylene spacer occurs within less than 10 ns in CH_2_Cl_2_ at 22 °C (*k*_ET_ ≥ 10^8^ s^–1^). Under these conditions, two fluoride anions bind to the organoboron unit with association constants greater than 10^5^ M^–1^ due to strong Lewis base/Lewis acid interactions.^5^ With two F^–^ anions bound to the 2,5-diboryl-1,4-phenylene spacer, the abovementioned photoinduced electron transfer process occurs with a time constant longer than 1000 ns (*k*_ET_ < 10^6^ s^–1^). The decrease of electron transfer rates by more than two orders of magnitude is caused by an increase of the tunneling barrier height upon fluoride binding (Scheme 3),31*a* leading to a decrease of the electronic interaction between the donor and the acceptor as the bridge LUMO is shifted to higher energy;1a,^38^ the organoboron bridging unit in its initial form is a strong π-acceptor, but the organofluoroborate species resulting from F^–^ addition has a much weaker π-acceptor capacity.22*a* In a more simplistic view, the bound F^–^ anions act as a Coulomb barrier for the transferring electron.^39^ Importantly, the driving force for electron transfer is essentially unaffected, unlike in the vast majority of previously explored examples in which chemical stimuli interact either with the donor or the acceptor to modulate the driving force for electron transfer (Δ*G*0ET).^10^–^12^ In our case the chemical stimulus (F^–^) interacts exclusively with the molecular bridge, and, to the best of our knowledge, our system is the first example of an anion-controllable rigid rod-like molecular wire.^13^ The use of strong Lewis acid/Lewis base interactions to regulate electron transfer across a rigid rod-like molecular wire (without affecting the driving force) is conceptually novel.14a–g

Our results are relevant in the greater context of a future molecular electronics technology because they demonstrate the basic principle of anion-responsive molecular wires which could become part of nanoscopic electrical circuits. Based on our electron transfer study, the anion-triggered switching between conducting and insulating states of such wires has become a realistic goal. For this purpose rigid rod-like molecular structures are highly desirable. In our study, the electron transfer occurs over a distance of ∼15 Å but in principle the functional 2,5-diboryl-1,4-phenylene element can also be incorporated into longer wires.

Finally we note that our findings are also relevant in a biochemical context. In some proteins, electron transfer between distant redox partners occurs along a pathway containing charged amino acid side chains.^38^,^40^ Interactions of the latter with counter-ions will affect the electron density at the respective amino acids and hence will influence the strength of the electronic coupling between the donor and the acceptor.15*b* Thus, it is conceivable that Nature uses ion-responsive structural elements acting in a similar fashion as the organoboron bridge investigated herein.15*a* Our study shows that this particular type of electron transfer rate regulation can be very effective.

## Supplementary Material

Supplementary information
