# Supplementary material for: Fluoride binding to an organoboron wire controls photoinduced electron transfer
Source: Chem Sci. 2015 May 1;6(6):3582–92. doi: 10.1039/c5sc00964b (PMC5659175; doi:10.1039/c5sc00964b)
Supplement: Supplementary file 1 [file SC-006-C5SC00964B-s001.pdf]

Electronic Supplementary Material (ESI) for Chemical Science.
This journal is © The Royal Society of Chemistry 2015

Supporting Information for:

Fluoride Binding to an Organoboron Wire Controls Photoinduced Electron Transfer

Jing Chen† and Oliver S. Wenger*

Department of Chemistry, University of Basel, St. Johanns-Ring 19, 4056 Basel, Switzerland

*Corresponding author. Email: oliver.wenger@unibas.ch

†Current address: Xiamen Institute of Rare-Earth Materials, Chinese Academy of Sciences, Amoy Institute of Technovation, Jimei Avenue 1300, Jimei Distric, Xiamen, Fujian, 361024, People's Republic of China

Table of contents

Methods and equipment S2

Synthesis and product characterization data S3

UV-Vis fluoride titration for determination of association constants S13

^{19}F and ^{11}B NMR spectra S16

Luminescence of **TAA-B-Ru^{2+}**, **B-Ru^{2+}**, and $Ru(bpy)_3^{2+}$ in CH_2Cl_2 in absence of fluoride S18

Transients for **TAA-BF$_2^-$-Ru^{2+}** and **BF$_2^-$-Ru^{2+}** in CH_2Cl_2 S20

Additional optical spectroscopic data for **B-Ru^{2+}** and $Ru(bpy)_3^{2+}$ in absence and presence of F^- S21

Cyclic voltammetry in presence of fluoride S26

References S27

Methods and equipment

^{1}H and ^{13}C NMR spectra were measured on a Bruker Avance III instrument operating at 400 or 600 MHz proton frequency. The instrument was equipped with a direct observe 5-mm BBFO smart probe. ^{11}B and ^{19}F spectra were recorded in deuterated dichloromethane at 192.55 and 564.61 MHz accumulating 128 and 32 scans, respectively. Both heteronuclear experiments were recorded with ^{1}H broad-band decoupling using a waltz16 sequence. For ^{19}F a baseline correction was applied using an identical NMR tube and identical solvent but without any dissolved compound. The resulting blank spectrum was subtracted from the sample spectrum to compensate for the broad solid-state background F resonance from Teflon parts in the probe head. An exponential window function with a coefficient of 5.0 and 100.0 Hz was used to process the ^{19}F and ^{11}B data, respectively. Fluoride addition occurred in the form of commercial 1.0 M tetra-*n*-butylammonium fluoride (TBAF) solution in THF. Mass spectra were recorded on a Bruker esquire 3000 plus or on a Bruker maxis 4G QTOF ESI spectrometer. Elemental analysis was measured by Ms. Sylvie Mittelheisser (Department of Chemistry, University of Basel) with a Varia Micro Cube instrument from Elementar. A Cary 5000 UV-Vis-NIR spectrophotometer from Varian was employed for optical absorption spectroscopy. Steady-state luminescence experiments were performed using a Fluorolog-322 instrument from Horiba Jobin-Yvon. Luminescence decays and transient absorption were measured on an LP920-KS spectrometer from Edinburgh Instruments, using the frequency-doubled output of a Quantel Brilliant b Nd:YAG laser for excitation. The laser pulse duration was approximately 10 ns, the pulse frequency was 10 Hz. Detection occurred either with an iCCD camera from Andor or an R928 photomultiplier tube. Transient difference spectra were time-averaged over the duration of 200 ns. Quartz cuvettes from Starna were employed for all optical spectroscopic studies; the cuvettes were fused to appropriate glassware in order to permit thorough de-oxygenation via the freeze-pump-thaw technique (3 cycles). Cyclic voltammetry was performed in a conventional setup with three electrodes using a Versastat3-200 potentiostat from Princeton Applied Research. A glassy carbon disk was used as a working electrode, and two silver wires served as counter and quasi-reference electrodes, respectively. Internal voltage calibration occurred by addition of small amounts of decamethylferrocene.[1] Dry CH_2Cl_2 with

0.1 M tetra-*n*-butylammonium hexafluorophosphate ($TBAPF_6$) was used as a solvent for all electrochemical measurements. Spectroelectrochemistry occurred with a Pt grid electrode immersed into a suitable cuvette.

Synthesis and product characterization data

Short overview

The **TAA-B-Ru^{2+}** and **B-Ru^{2+}** compounds each contain a functionalized bpy (bpy = 2,2'-bipyridine) ligand which can be synthesized as illustrated by Scheme S1 (identical to Scheme 2 in the main paper). 2,5-Dibromoaniline (**1**) was reacted with *N*-iodosuccinimide to yield 2,5-dibromo-4-iodoaniline (**2**).[2] Molecule (**2**) was converted to a diethyltriazine (**3**),[3] and subsequent reaction with trimethylsilylacetylene afforded molecule (**4**). The latter was reacted with *n*-butyllithium and dimesitylfluoroborane to afford bis(dimesitylboryl) compound **5**.[4] Reaction with methyliodide in a sealed tube then gave the doubly dimesitylboryl-decorated ((4-iodophenyl)ethynyl)trimethylsilane **6**.[5] Compound **6** resembles building blocks which were previously employed for the modular synthesis of oligo(*p*-phenylene ethynylene) (OPE) "wires" with the important novelty that compound **6** is equipped with two lateral dimesitylboron units.[6] Compound **6** can be produced from starting material **1** in 9% overall yield.

The triarylamino-group was incorporated into the overall system by coupling commercially available dianisylamine (**7**) to bromobenzene (**8**) and subsequent reaction of the coupling product (**9**) with *N*-iodosuccinimide to afford 4-iodo-*N,N*-bis(4-methoxyphenyl)aniline (**10**).[7] The latter was reacted with trimethylsilylacetylene, and the coupling product (**11**) was deprotected with KOH in a mixture of methanol and CH_2Cl_2 to give molecule **12** in 50% overall yield with respect to the dianisylamine starting material (**7**).

Compound **12** and compound **6** were then reacted under standard Sonogashira coupling conditions. Deprotection of the coupling product (**13**) afforded molecule **14**, which was coupled to 5-bromo-2,2'-

bipyridine (**15**)[8] to yield the final ligand (**16**). Its coordination to Ru(bpy)$_2$Cl$_2$ gave the **TAA-B-Ru^{2+}** dyad.

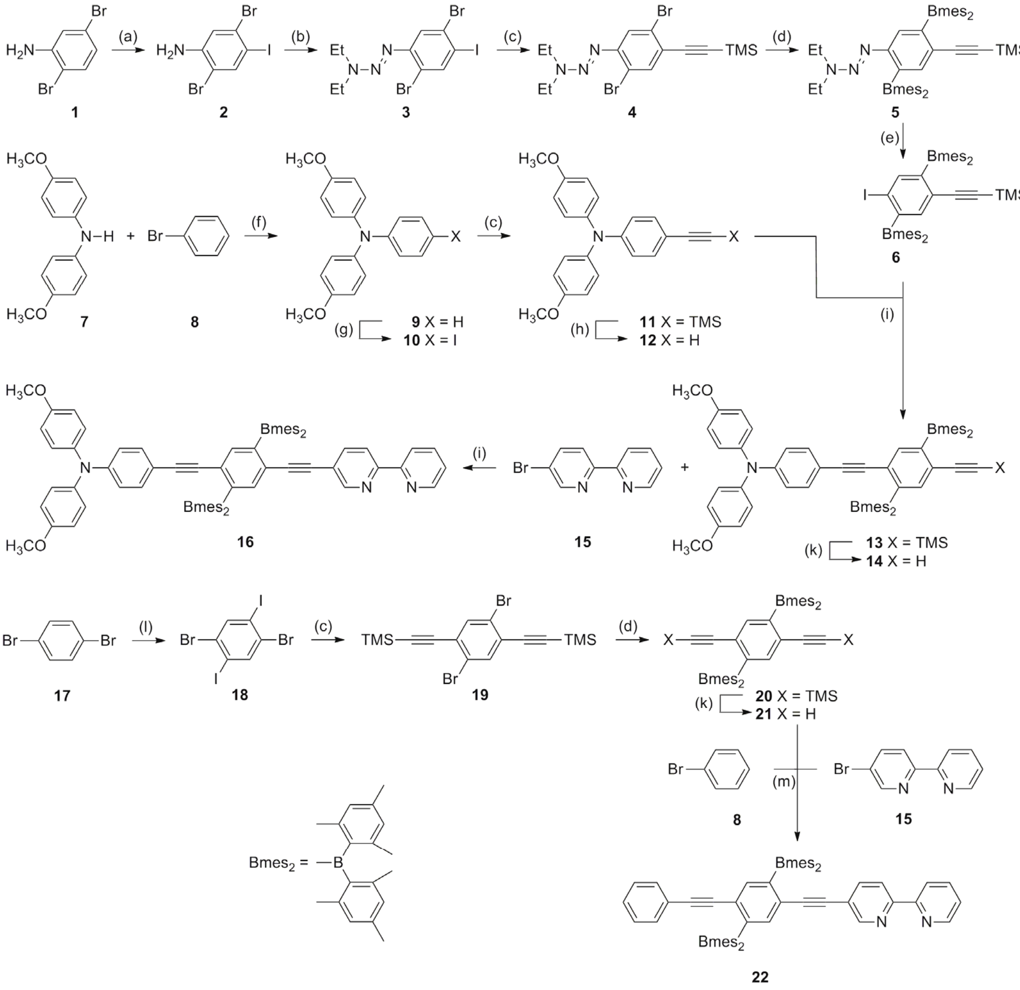

Scheme S1. Synthesis of the key ligands. (a) NIS, DMSO; (b) BF$_3$·Et$_2$O, ^{t}BuONO, CH$_2$Cl$_2$, Et$_2$NH; (c) TMS-C≡C-H, CuI, Pd(PPh$_3$)$_2$Cl$_2$, Et$_3$N, THF; (d) *n*-BuLi, Bmes$_2$F, Et$_2$O; (e) MeI; (f) P(^{t}Bu)$_3$H$^+$BF$_4^-$, Pd(dba)$_2$, ^{t}BuOK, toluene; (g) NIS, DMF; (h) KOH, MeOH, CH$_2$Cl$_2$; (i) Pd(PPh$_3$)$_4$, CuI, THF, ^{i}Pr$_2$NH; (k) NaH, THF; (l) I$_2$, conc. H$_2$SO$_4$; (m) Pd(PPh$_3$)$_4$, CuI, Et$_3$N. (This Scheme is identical to Scheme 1 in the main paper).

Synthesis of the **B-Ru^{2+}** reference compound (chemical structure shown in Scheme 1c of the main paper) departed from 1,4-dibromobenzene (**17**) which was converted to 1,4-diodo-2,5-dibromobenzene (**18**).[9] Coupling to trimethylsilylacetylene afforded compound **19**. The latter was

reacted with *n*-butyllithium and commercial dimesitylfluoroborane to give the symmetrical bis(dimesitylboron)-substituted building block **20**. Deprotection of its two trimethylsilyl-groups occurred with NaH in THF to afford molecule **21**.[4] The latter was reacted with 1 equivalent of bromobenzene (**8**) and 1 equivalent of 5-bromo-2,2'-bipyridine (**15**)[8] under standard Sonogashira coupling conditions, yielding final ligand **22**. Its coordination to $Ru(bpy)_2Cl_2$ afforded the **B-Ru^{2+}** reference molecule.

Full synthetic details

2,5-dibromo-4-iodoaniline (**2**). Following a previously published protocol,[2] commercial 2,5-dibromoaniline (**1**) (5.12 g, 20 mmol) and *N*-iodosuccinimide (4.52 g, 20 mmol) were dissolved in dry DMSO (80 ml). The reaction mixture was stirred at room temperature for 3 days. Then, saturated aqueous $NaHCO_3$ solution (200 ml) was added, followed by extraction with EtOAc (3×300 ml). The combined organic phases were washed with brine and dried over anhydrous Na_2SO_4. Solvent removal with a rotary evaporator afforded a gray solid which was dried in vacuum and further purified with column chromatography on silica gel, using a 9:1 (v:v) mixture of CH_2Cl_2 and cyclohexane as the eluent. This afforded the product as a grey solid (7.1 g, 94%). ^{1}H NMR ($CDCl_3$, 400 MHz) δ = 7.77 (s, 1 H), 7.01 (s, 1 H), 4.16 (s, 2 H). ^{13}C NMR ($CDCl_3$, 101 MHz) δ = 145.2, 141.9, 128.9, 118.6, 108.6, 85.3. ESI-MS (m/z): calc. for $C_6H_4NBr_2I$: 376.8, found: 377.8 (M+H$^+$).

1-(2,5-dibromo-4-iodophenyl)-3,3-diethyltriazine (**3**). To a solution of 2,5-dibromo-4-iodoaniline (**2**) (565 mg, 1.5 mmol) in CH_2Cl_2 at -20 °C was added boron trifluoride etherate (0.38 ml, 3 mmol), followed by *t*-butyl nitrite (0.36 ml, 3 mmol).[3a] The reaction mixture was stirred at -20 °C for 30 minutes and then at 0 °C for another 30 minutes. Subsequently, diethylamine (1.24 ml, 12 mmol) and K_2CO_3 (1.38 g, 10 mmol) were added, and the mixture was stirred at 0 °C for 2 hours. After that, water (100 ml) was added, and the organic phase was separated. The aqueous layer was extracted with ethyl acetate (3×50 ml) and the combined organic phases were dried over anhydrous Na_2SO_4. After solvent

removal, the crude product was purified by column chromatography on silica gel using a CH_2Cl_2 / cyclohexane 1:2 (v:v) mixture as the eluent. This afforded the desired product as a yellow solid (660 mg, 96%). ^{1}H NMR ($CDCl_3$, 400 MHz) δ = 8.01 (s, 1 H), 7.65 (s, 1 H), 3.79 (q, *J* = 7.1 Hz, 4 H), 1.34 (t, *J* = 7.1 Hz, 3 H), 1.26 (t, *J* = 7.1 Hz, 3 H). ^{13}C NMR ($CDCl_3$, 101 MHz) δ = 149.4, 142.8, 128.9, 121.7, 118.9, 95.0, 49.7, 42.5, 14.6, 10.8. ESI-MS (m/z): calc. for $C_{10}H_{12}N_3Br_2I$: 460.8, found: 462.1 ($M+H^+$).

1-(2,5-dibromo-4-((trimethylsilyl)ethynyl)phenyl)-3,3-diethyltriazine (**4**). Molecule **3** (200 mg, 0.43 mmol) and trimethylsilylacetylene (63 µl, 0.44 mmol) were dissolved in a mixture of triethylamine (5 ml) and THF (5 ml). The solution was de-oxygenated with three freeze-pump-thaw cycles and then CuI (8.3 mg, 10 mol-%) and $PdCl_2(PPh_3)_2$ (15 mg, 5 mol-%) were added. This mixture was stirred at 60 °C for 24 hours. Completeness of the reaction was verified by thin layer chromatography. The solvents were removed on a rotary evaporator. Column chromatography on silica gel with a mixture of CH_2Cl_2 and cyclohexane (1:9, v:v) gave the product as a yellow solid (155 mg, 83%).^{1}H NMR ($CDCl_3$, 400 MHz) δ = 7.70 (s, 1 H), 7.63 (s, 1 H), 3.80 (q, *J* = 7.0 Hz, 4 H), 1.79 (t, *J* = 7.0 Hz, 3 H), 1.27 (t, *J* = 7.0 Hz, 3 H), 0.28 (s, 9 H). ^{13}C NMR ($CDCl_3$, 101 MHz) δ = 149.1, 137.3, 125.0, 121.5, 117.9, 102.2, 100.0, 49.8, 42.6, 14.6, 10.9, 0.0. ESI-MS (m/z): calc. for $C_{15}H_{21}N_3Br_2Si$: 431.0, found: 432.2 ($M+H^+$).

1-(2,5-bis(dimesitylboryl)-4-((trimethylsilyl)ethynyl)phenyl)-3,3-diethyltriazine (**5**). Following a previously published protocol,[4] molecule **4** (206 mg, 0.48 mmol) was dissolved in dry diethyl ether (4 ml) and cooled to -78 °C under N_2 atmosphere. Then, 1.6 M *n*-butyllithium in hexane (0.63 ml, 1.00 mmol) was added drop by drop, and the reaction mixture was stirred at -78 °C for 30 minutes before allowing it to warm up to room temperature. After stirring for 1 hour at room temperature it was cooled again to -78 °C and a solution of dimesitylfluoroborane (275 mg, 1.00 mmol) in diethyl ether (2 ml) was added slowly. The mixture was allowed to warm up to room temperature while stirring overnight. The product appeared as a yellow precipitate. The latter was filtered, washed with diethyl ether and dried in vacuum (148 mg, 40 %). ^{1}H NMR ($CDCl_3$, 400 MHz) δ = 7.34 (s, 1 H), 7.20 (s, 1

H), 6.74 (s, 4 H), 6.69 (s, 4 H), 3.30 (s, 4 H), 2.26 (s, 6 H), 2.23 (s, 6 H), 2.02 (s, 12 H), 1.95 (s, 12 H), 0.85 (s, 6 H), -0.06 (s, 9 H). ^{13}C NMR ($CDCl_3$, 101 MHz) δ = 154.4, 141.1, 140.7, 139.0, 138.8, 138.4, 128.4, 128.3, 124.8, 122.7, 106.1, 96.2, 23.8, 23.5, 21.4, 21.3, 0.0. ESI-MS (m/z): calc. for $C_{51}H_{65}N_3B_2Si$: 769.5, found: 770.8 ($M+H^+$).

((2,5-bis(dimesitylboryl)-4-iodophenyl)ethynyl)trimethylsilane (**6**). Following a previously published method,[5] molecule **5** (200 mg, 0.26 mmol) and methyl iodide (0.9 ml, 14.5 mol) were sealed into a tube under N_2 atmosphere and heated to 130 °C for 24 hours. After cooling to room temperature, saturated aqueous ammonia solution (30 ml) was added. After extracting with CH_2Cl_2 (3×30 ml), the combined organic phases were dried over anhydrous Na_2SO_4. After solvent removal, the crude product was purified by column chromatography on silica gel. The eluent was a 1:2 (v:v) mixture of CH_2Cl_2 and cyclohexane. This procedure yielded the pure product (80 mg, 30%). ^{1}H NMR ($CDCl_3$, 400 MHz) δ = 7.67 (s, 1 H), 7.22 (s, 1 H), 6.77 (s, 4 H), 6.75 (s, 4 H), 2.29 (s, 6 H), 2.27 (s, 6 H), 1.99 (s, 24 H), -0.06 (s, 9 H). ^{13}C NMR ($CDCl_3$, 101 MHz) δ = 155.2, 154.6, 141.0, 140.0, 139.6, 139.3, 128.6, 128.5, 126.0, 104.3, 102.4, 98.9, 23.5, 21.5, 21.4, -0.2. ESI-MS (m/z): calc. for $C_{47}H_{55}B_2ISi$: 796.3, found: 797.7 ($M+H^+$).

N,*N*-bis(4-methoxyphenyl)-phenylaniline (**9**). To a flask containing 4,4'-dimethoxydiphenylamine (**7**) (4.58 g, 20 mmol), bromobenzene (**8**) (2.1 ml, 20 mmol), tri-*t*-butylphosphonium tetrafluoroborate (232 mg, 0.8 mmol), ^{t}BuOK (3.34 g, 30 mmol), and bis(dibenzylideneacetone)palladium (464 mg, 0.8 mmol) under N_2, dry toluene (200 ml) was added.[10] The reaction mixture was heated to 80 °C for 24 hours. Water was added to quench the reaction and the mixture was extracted with CH_2Cl_2. After drying the combined organic phases over anhydrous Na_2SO_4 the solvent was evaporated, and the resulting oil was chromatographed on silica gel, using a mixture of CH_2Cl_2 and cyclohexane (1:2, v:v) as the eluent. The product was obtained as a white solid (5.5 g, 90 %). ^{1}H NMR ($CDCl_3$, 400 MHz) δ = 7.19-7.15 (m, 2 H), 7.06-7.03 (m, 4 H), 6.94 (d, J = 7.9 Hz, 2 H), 6.88-6.80 (m, 5 H), 3.79 (s, 6 H). ^{13}C NMR ($CDCl_3$, 101 MHz) δ = 155.8, 148.9, 141.3, 129.1, 126.5, 121.1, 120.7, 114.8, 55.6. ESI-MS (m/z): calc. for $C_{20}H_{19}NO_2$: 305.1, found: 305.2 (M^+).

4-iodo-*N,N*-bis(4-methoxyphenyl)aniline (**10**). Following a previously published method,[7] molecule **9** (1.95 g, 6.4 mmol) and *N*-iodosuccinimide (1.5 g, 6.5 mmol) were dissolved in DMF (10 ml) and stirred for 3 hours at room temperature. After complete consumption of the starting materials as verified by thin layer chromatography, the reaction was quenched by addition of aqueous $NaHCO_3$ solution. After extraction with CH_2Cl_2 the combined organic phases were dried over anhydrous Na_2SO_4. Solvent removal under reduced pressure yielded the product as a yellow solid (2.1 g, 76 %). ^{1}H NMR ($CDCl_3$, 400 MHz) δ = 7.41 (d, *J* = 8.6 Hz, 2 H), 7.01 (d, *J* = 8.6 Hz, 4 H), 6.85 (m, 4 H), 6.71 (d, *J* = 8.6 Hz, 2 H), 3.79 (s, 6 H). ^{13}C NMR ($CDCl_3$, 101 MHz) δ = 156.2, 148.6, 140.4, 137.7, 126.8, 122.3, 114.9, 82.1, 56.5. ESI-MS (m/z): calc. for $C_{20}H_{18}NIO_2$: 431.0, found: 432.2 ($M+H^+$).

N,*N*-bis(4-methoxyphenyl)-*N*-(4-trimethylsilylethynylphenyl)amine (**11**). Molecule **10** (1.6 g, 3.7 mmol) and trimethylsilylacetylene (0.55 ml, 3.8 mmol) were dissolved in a mixture of triethylamine (20 ml) and THF (40 ml). After de-oxygenating with three freeze-pump-thaw cycles, CuI (70 mg, 10 mol-%) and $PdCl_2(PPh_3)_2$ (130 mg, 5 mol-%) were added. The reaction mixture was stirred at 60 °C for 24 hours, and then the solvents were removed under reduced pressure. Column chromatography on silica gel using a CH_2Cl_2 / cyclohexane (1:2, v:v) mixture as the eluent gave the product as a yellow solid (1.34 g, 90 %). ^{1}H NMR ($CDCl_3$, 400 MHz) δ = 7.25-7.23 (m, 2 H), 7.06-7.02 (m, 4 H), 6.86-6.77 (m, 6 H), 3.80 (s, 6 H), 0.23 (s, 9 H). ^{13}C NMR ($CDCl_3$, 101 MHz) δ = 155.4, 148.1, 139.4, 131.9, 126.2, 118.2, 113.9, 113.0, 105.0, 91.4, 54.6, -0.7.

N,*N*-bis(4-methoxyphenyl)-*N*-(4-ethynylphenyl)amine (**12**). Molecule **11** (1.34 g, 3.34 mmol) was dissolved in a mixture of methanol (35 ml) and CH_2Cl_2 (15 ml) under N_2 atmosphere. KOH (0.19 g, 3.4 mmol) was added, and the mixture was stirred at room temperature for 2.5 hours. After solvent removal under reduced pressure, the crude product was purified on a silica gel column using a 1:1 (v:v) mixture of CH_2Cl_2 and cyclohexane as the eluent. This afforded the product as a brownish solid (0.91 g, 81 %). ^{1}H NMR ($CDCl_3$, 400 MHz) δ = 7.28-7.25 (m, 2 H), 7.06-7.04 (m, 4 H), 6.84-6.78 (m,

6 H), 3.79 (s, 6 H), 3.33 (s, 1 H). ^{13}C NMR ($CDCl_3$, 101 MHz) δ = 156.5, 149.3, 140.2, 133.0, 127.3, 119.0, 114.9, 112.7, 84.4, 75.7, 55.6. ESI-MS (m/z): calc. for $C_{22}H_{19}NO_2$: 329.1, found: 329.2 (M^+).

Molecule **13**. To a flask containing *N*,*N*-bis(4-methoxyphenyl)-*N*-(4-ethynylphenyl)amine (**12**) (64 mg, 0.19 mmol), molecule **6** (140 mg, 0.18 mmol), CuI (3.8 mg, 0,02 mmol), and $Pd(PPh_3)_4$ (12 mg, 0.01 mmol) under N_2 were added THF (9 ml) and diisopropylamine (3 ml). The reaction mixture was bubbled with N_2 gas for a few minutes prior to heating to 80 °C for 24 hours. Then the solvents were removed under reduced pressure and the crude product was purified by column chromatography on silica gel. The eluent was a 1:2 (v:v) mixture of CH_2Cl_2 and cyclohexane. The final product was washed with acetonitrile to afford an orange solid (54 mg, 31 %). ^{1}H NMR ($CDCl_3$, 400 MHz) δ = 7.37 (s, 1 H), 7.34 (s, 1 H), 7.02-6.98 (m, 4 H), 6.82-6.77 (m, 6 H), 6.74 (s, 8 H), 6.69-6.65 (m, 2 H), 3.79 (s, 6 H), 2.27 (s, 6 H), 2.24 (s, 6 H), 2.01 (s, 24 H). ESI-MS (m/z): calc. for $C_{69}H_{73}NO_2B_2Si$: 997.6, found: 999.0 ($M+H^+$).

Molecule **14**. Molecule **13** (500 mg, 0.5 mmol) and NaH (24 mg, 0.6 mmol) were added to dry THF (80 ml), and the mixture was stirred at 80 °C for 9 hours. After solvent removal under reduced pressure, the raw product was purified by column chromatography on silica gel with a 1:2 (v:v) mixture of CH_2Cl_2 and cyclohexane as the eluent. The product was obtained as an orange solid (230 mg, 50 %). ^{1}H NMR ($CDCl_3$, 400 MHz) δ = 7.42 (s, 1 H), 7.40 (s, 1 H), 7.04 (d, *J* = 8.8 Hz, 4 H), 6.85-6.75 (m, 14 H), 6.69 (d, *J* = 8.6 Hz, 2 H), 3.80 (s, 6 H), 2.74 (s, 1 H), 2.29 (s, 6 H), 2.25 (s, 6 H), 2.04 (s, 12 H), 2.03 (s, 12 H). ^{13}C NMR ($CDCl_3$, 101 MHz) δ = 156.3, 153.3, 148.6, 142.6, 141.1, 141.0, 140.5, 139.5, 139.3, 138.3, 137.4, 132.6, 128.5, 128.3, 127.2, 127.0, 124.1, 119.2, 114.9, 114.2, 95.0, 89.2, 83.6, 81.2, 55.6, 23.4, 23.4, 21.4, 21.3.

Ligand **16**. Molecule **14** (160 mg, 0.173 mmol), 5-bromo-2,2'-bipyridine (**15**)[8] (40 mg, 0.170 mmol), CuI (5 mg, 0.026 mmol), and $Pd(PPh_3)_4$ (15 mg, 0.013 mmol) were added to de-oxygenated mixture of THF (18 ml) and diisopropylamine (6 ml). The reaction mixture was heated to 80 °C for 24 hours. After solvent removal, chromatography on a silica gel column with an eluent comprised of a

100:100:2 (v:v:v) mixture of CH_2Cl_2, cyclohexane, and triethylamine gave an orange solid. After washing with acetonitrile the pure product (140 mg, 77 %) was obtained. ^1H NMR ($CDCl_3$, 400 MHz) δ = 8.68 (d, J = 3.9 Hz, 1 H), 8.41 (d, J = 7.8 Hz, 1 H), 8.33 (s, 1 H), 8.27 (d, J = 8.1 Hz, 1 H), 7.83 (t, J = 7.3 Hz, 1 H), 7.47 (s, 1 H), 7.45 (s, 1 H), 7.36 (dd, J = 8.1, 2.0 Hz, 1 H), 7.34-7.29 (m, 1 H), 7.06-6.98 (m, 4 H), 6.85-6.80 (m, 6 H), 6.77 (s, 8 H), 6.70-6.68 (m, 2 H), 3.80 (s, 6 H), 2.26 (s, 6 H), 2.25 (s, 6 H), 2.05 (s, 24 H). ESI-HRMS (m/z): calc. for $C_{76}H_{71}N_3O_2B_2$: 1079.5749, found: 1079.5734. Elemental analysis calc. for $C_{76}H_{71}N_3O_2B_2$+$3H_2O$ (%): C, 80.49; H, 6.84; N, 3.71. Found: C, 80.82; H, 6.94; N, 3.62.

Dyad **TAA-B-Ru^{2+}**. $Ru(bpy)_2Cl_2$ (54 mg, 0.11 mmol) and ligand **16** (120 mg, 0.11 mmol) were heated to reflux in a mixture of chloroform (12 ml) and ethanol (12 ml) overnight. Then the solvents were removed on a rotary evaporator. Column chromatography on silica gel occurred with a 100:10:1 (v:v:v) mixture of acetone, water and saturated aqueous KNO_3 solution. Acetone was evaporated from the desired chromatography fractions, and the product was precipitated by adding saturated aqueous KPF_6 solution. The orange solid was collected by filtration and was washed with de-ionized water and diethyl ether. After drying in vacuum the pure product was isolated (150 mg, 77 %). ^1H NMR (acetone-d_6, 400 MHz) δ = 8.82-8.76 (m, 6 H), 8.29-8.19 (m, 5 H), 8.09-8.03 (m, 4 H), 7.98 (d, J = 5.4 Hz, 1 H), 7.79 (s, 1 H), 7.67-7.50 (m, 6 H), 7.44 (s, 1 H), 7.06 (d, J = 8.8 Hz, 4 H), 6.92 (d, J = 8.8 H, 4 H), 6.86 (s, 8 H), 6.79 (d, J = 8.6 Hz, 2 H), 6.62 (d, J = 8.6 Hz, 2 H), 6.54 (s, 1 H), 3.80 (s, 6 H), 2.25 (s, 6 H), 2.05 (s, 18 H), 1.94 (s, 12 H). ^1H NMR (CD_2Cl_2, 600 MHz) δ = 8.47-8.40 (m, 4 H), 8.34 (d, J = 8.1 Hz, 1 H), 8.27 (d, J = 8.5 Hz, 1 H), 8.10-8.05 (m, 4 H), 7.95 (t, J = 7.8 Hz, 1 H), 7.69-7.60 (m, 5 H), 7.52-7.40 (m, 6 H), 7.34 (t, J = 6.5 Hz, 1 H), 7.28-7.26 (m, 2 H), 7.02 (d, J = 8.7 Hz, 4 H), 6.84 (d, J = 8.8 Hz, 4 H), 6.79-6.64 (m, 12 H), 3.78 (s, 6 H), 2.26 (s, 6 H), 2.22 (s, 6 H), 1.99 (s, 12 H), 1.95 (s, 12 H). ^{13}C NMR (acetone-d_6, 101 MHz) δ = 158.2, 158.1, 158.0, 157.8, 157.5, 156.8, 153.7, 153.1, 153.0, 152.8, 152.6, 150.2, 141.5, 140.6, 140.5, 140.3, 139.2, 139.1, 139.0, 138.8, 138.4, 138.3, 133.3, 129.5, 129.4, 129.0, 128.9, 128.8, 128.7, 128.6, 125.8, 125.6, 125.4, 125.3, 125.2, 124.6, 124.5, 124.3, 118.6, 115.8, 113.5, 97.7, 97.3, 89.3, 89.1, 55.8, 21.4, 21.3. ESI-HRMS (m/z): calc. for

$C_{96}H_{87}N_7O_2B_2Ru^{2+}$: 746.8074, found: 746.8092. Elemental analysis calc. for $C_{96}H_{87}N_7O_2B_2F_{12}P_2Ru{\cdot}2H_2O$ (%): C, 63.37; H, 5.04; N, 5.39. Found: C, 63.19; H, 5.42; N, 5.36.

1,4-dibromo-2,5-diiodobenzene (**18**). This compound was prepared from 1,4-dibromobenzene (**17**) as described previously.[9]

1,4-dibromo-2,5-bis(trimethylsilylethynyl)benzene (**19**). 1,4-dibromo-2,5-diiodobenzene (**18**)[9] (2.0 g, 4.1 mmol) and trimethylsilylacetylene (1.2 ml, 8.2 mol) were dissolved in a mixture of triethylamine (30 ml) and THF (30 ml). After de-oxygenating the solution with three freeze-pump-thaw cycles, CuI (156 mg, 10 mol-%) and $PdCl_2(PPh_3)_2$ (302 mg, 5 mol-%) were added. The reaction mixture was heated to 80 °C for 24 hours, then the solvent was removed under reduced pressure. Chromatography on a silica gel column with cyclohexane gave the product as a white solid (1.5 g, 85%). ^{1}H NMR ($CDCl_3$, 400 MHz) δ = 7.67 (s, 2 H), 0.27 (s, 18 H). ^{13}C NMR ($CDCl_3$, 101 MHz) δ = 136.6, 126.6, 123.9, 103.2, 101.5, -0.2.

1,4-bis(dimesitylboryl)-2,5-bis(trimethylsilylethynyl)benzene (**20**). 1,4-dibromo-2,5-bis(trimethylsilylethynyl)benzene (**19**) (545 mg, 1.27 mmol) was dissolved in dry diethyl ether (10 ml) and the mixture was cooled to -78 °C. Then, 2.5 M *n*-butyllithium in hexane (1.02 ml, 2.55 mmol) was added drop by drop, and the mixture was stirred for 30 minutes at -78 °C. After stirring at room temperature for 1 hour, the reaction mixture was cooled again to -78 °C and dimesitylboron fluoride (700 mg) in diethyl ether (5 ml) was added. The mixture was allowed to warm up to room temperature overnight while stirring. The resulting precipitate was filtered and washed with diethyl ether, yielding the product as a white solid (0.85 g, 87 %). ^{1}H NMR ($CDCl_3$, 400 MHz) δ = 7.34 (s, 2 H), 6.75 (s, 8 H), 2.28 (s, 12 H), 1.99 (s, 24 H), -0.06 (s, 18 H). ^{13}C NMR ($CDCl_3$, 101 MHz) δ = 152.8, 142.6, 141.1, 139.4, 137.5, 128.5, 125.9, 105.0, 99.2, 23.5, 21.4, -0.2.

1,4-bis(diethynyl)-2,5- bis(dimesitylboryl)benzene (**21**). Molecule **20** (300 mg, 0.39 mmol) and NaH (94 mg, 2.35 mmol) were dispersed in dry THF (40 ml). After heating to 80 °C for 7 hours, the solvent

was removed. Column chromatography on silica gel with a 1:2 (v:v) mixture of CH_2Cl_2 and cyclohexane gave the product (228 mg, 94 %) as a white solid. ^{1}H NMR ($CDCl_3$, 400 MHz) δ = 7.39 (s, 2 H), 6.75 (s, 8 H), 2.73 (s, 2 H), 2.27 (s, 12 H), 2.00 (s, 24 H). ^{13}C NMR ($CDCl_3$, 101 MHz) δ = 153.2, 142.5, 141.1, 139.6, 137.9, 128.3, 125.2, 83.2, 81.7, 23.3, 21.4.

Ligand **22**. Molecule **21** (220 mg, 0.35 mmol), 5-bromo-2,2'-bipyridine (**15**) (82 mg, 0.35 mmol), bromobenzene (**8**) (73 µl, 0.35 mmol), CuI (7 mg, 10 mol-%), and $Pd(PPh_3)_4$ (21 mg, 5 mol-%) were added to triethylamine (20 ml) under N_2. The reaction mixture was heated to 80 °C for 36 hours. Then the solvents were removed and purification occurred by chromatography on a silica gel column with a 50:50:1 (v:v:v) mixture of CH_2Cl_2, cyclohexane and triethylamine as the eluent. This afforded the pure product (58 mg, 19%). ^{1}H NMR ($CDCl_3$, 400 MHz) δ = 8.60 (d, *J* = 4.0 Hz, 1 H), 8.32 (d, *J* = 8.0 Hz, 1 H), 8.26 (d, *J* = 8.0 Hz, 1 H), 8.18 (d, *J* = 8.3 Hz, 1 H), 7.75 (td, *J* = 8.0, 1.6 Hz, 1 H), 7.42 (d, *J* = 1.3 Hz, 1 H), 7.28 (dt, *J* = 8.2, 2.0 Hz, 1 H), 7.23 (dd, *J* = 6.5, 4.0 Hz, 1 H), 7.15-7.07 (m, 3 H), 6.93 (dd, *J* = 8.0, 1.3 Hz, 2 H), 6.70 (s, 8 H), 2.19 (s, 12 H), 1.98 (s, 24 H). ^{13}C NMR ($CDCl_3$, 101 MHz) δ = 155.7, 153.8, 152.4, 151.5, 149.0, 142.6, 141.1, 139.7, 139.5, 137.9, 137.8, 131.7, 128.6, 128.4, 128.1, 127.9, 126.7, 125.3, 124.1, 123.2, 121.8, 120.6, 120.1, 94.6, 94.3, 90.4, 90.2, 23.5, 21.4, 21.3. ESI-HRMS (m/z): calc. for $C_{62}H_{58}N_2B_2$: 853.4878, found: 853.4872.

Reference compound **B-Ru^{2+}**. $Ru(bpy)_2Cl_2$ (82 mg, 0.17 mmol) and ligand **22** (130 mg, 0.15 mmol) were heated to reflux in a mixture of chloroform (20 ml) and ethanol (20 ml) for 24 hours. Then the solvents were evaporated. Chromatography on a silica gel column occurred with a 100:10:1 (v:v:v) mixture of acetone, de-ionized water, and saturated aqueous KNO_3 solution. Acetone was evaporated from the desired chromatography fractions and saturated aqueous KPF_6 solution was added. The orange precipitate was filtered, washed with water and then dried under vacuum (100 mg, 42%). ^{1}H NMR (CD_3CN, 400 MHz) δ = 8.48 (m, 6 H), 8.37 (d, *J* = 8.5 Hz, 1 H), 8.12-8.02 (m, 5 H), 7.98 (td, *J* = 8.0, 1.3 Hz, 1 H), 7.69 (t, *J* = 4.9 Hz, 4 H), 7.64 (d, *J* = 5.1 Hz, 1 H), 7.53 (d, *J* = 1.4 Hz, 1 H), 7.48-7.21 (m, 12 H), 6.97 (dd, *J* = 8.1, 1.4 Hz, 2 H), 6.83 (s, 4 H), 6.76 (s, 1 H), 2.25 (s, 6 H), 2.20 (s, 15 H), 1.99 (s, 9 H), 1.90 (s, 6 H). ^{1}H NMR (CD_2Cl_2, 600 MHz) δ = 8.35-8.29 (m, 4 H), 8.24 (d, *J* = 7.6

Hz, 1 H), 8.16 (d, J = 8.3 Hz, 1 H), 7.97-7.93 (m, 4 H), 7.83 (t, J = 7.5 Hz, 1 H), 7.57-7.48 (m, 5 H), 7.39-7.32 (m, 6 H), 7.24-7.22 (m, 1 H), 7.18-7.16 (m, 2 H), 7.12-7.07 (m, 3 H), 6.85 (d, J = 7.3 Hz, 2 H), 6.68-6.63 (m, 8 H), 2.16 (s, 6 H), 2.10 (s, 6 H), 1.88 (s, 12 H), 1.83 (s, 12 H). ^{13}C NMR (CD_3CN, 101 MHz) δ = 157.9, 157.8, 157.3, 156.7, 153.7, 153.5, 153.1, 152.9, 152.8, 152.5, 143.1, 141.7, 141.0, 140.4, 139.0, 138.9, 138.8, 138.7, 138.4, 132.3, 129.7, 129.5, 129.4, 129.2, 128.8, 128.7, 128.6, 128.5, 128.0, 125.7, 125.5, 125.3, 125.2, 125.0, 124.5, 123.3, 97.4, 95.7, 90.1, 89.3, 23.5, 21.3, 21.2. ESI-HRMS (m/z): calc. for $C_{82}H_{74}N_6B_2Ru^{2+}$: 633.2616, found: 633.2616. Elemental analysis calc. for $C_{82}H_{74}N_6B_2F_{12}P_2Ru$+1$H_2O$ (%): C, 62.57; H, 4.87; N, 5.34. Found: C, 62.26; H, 4.90; N, 5.42.

UV-Vis fluoride titration for determination of association constants

For both **TAA-B-Ru^{2+}** and **B-Ru^{2+}** changes in the UV-Vis absorption spectrum are easily detectable upon addition of 1 – 4 equivalents of TBAF (tetra-*n*-butylammonium fluoride) to CH_2Cl_2 solutions of these compounds (dashed lines in Figure 1 of the main paper). Below are shown titration curves displaying the absorbance of CH_2Cl_2 solutions with known ($2\cdot10^{-5}$ M) **TAA-B-Ru^{2+}** (Figure S1) and **B-Ru^{2+}** concentrations (Figure S2) at selected wavelengths as a function F^- concentration. The two data sets were analyzed in terms of a 1:2 binding model using the Specfit software, i. e., it was assumed that two fluoride anions bind per **TAA-B-Ru^{2+}** or **B-Ru^{2+}** molecule because they both contain two dimesitylboron units. The obtained fits to the experimental titration curves are reasonably good (solid lines in Figures S1/S2), unlike what is obtained with a simpler 1:1 binding model (not shown). So-called component spectra used to obtain the fits with the 1:2 binding model are also included (Figure S3/S4). The cumulative binding constants for the formation of 1:1 ($\beta_{1,1}$) and 1:2 ($\beta_{1,2}$) adducts obtained in this manner are summarized in Table 1 of the main paper. In both **TAA-B-Ru^{2+}** and **B-Ru^{2+}** the first fluoride anion binds with an association constant (K_A) on the order of 10^7 M^{-1} while the second F^- binds with $K_A = 10^5 – 10^6$ M^{-1}. These values are in line with fluoride binding constants reported earlier for related organoboron compounds in similarly apolar solution.[11] A key point here is that dry CH_2Cl_2 must be used for accurate determination of association constants because F^- has a high hydration

enthalpy. As noted in the main paper, TBAF solution in THF usually contains some residual water, but this is difficult to avoid. We can exclude that any of the effects discussed in the following arises solely from the presence of water; fluoride is clearly the active species.

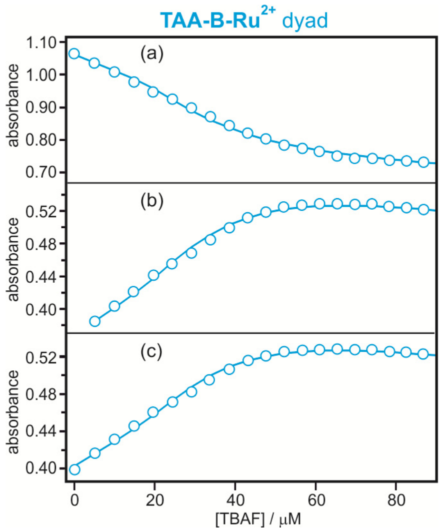

Figure S1. Open circles: Absorbance of a $2{\cdot}10^{-5}$ M solution of **TAA-B-Ru^{2+}** in CH_2Cl_2 at (a) 356 nm, (b) 425 nm, and (c) 440 nm as a function of nominal TBAF concentration. Solid lines: Fits obtained with a 1:2 (**TAA-B-Ru^{2+}** versus F^-) binding model using the K_A values reported in Table 1 of the main paper.

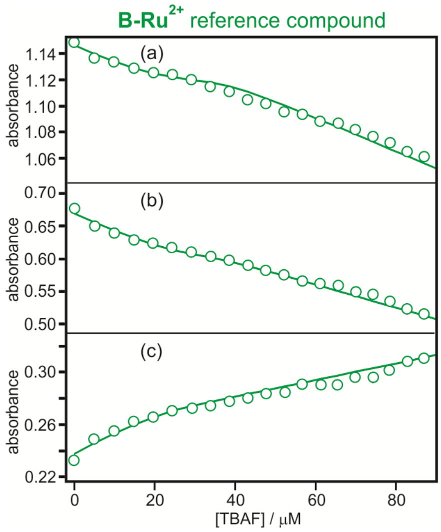

Figure S2. Open circles: Absorbance of a $2{\cdot}10^{-5}$ M solution of **B-Ru^{2+}** in CH_2Cl_2 at (a) 314 nm, (b) 380 nm, and (c) 470 nm as a function of nominal TBAF concentration. Solid lines: Fits obtained with a 1:2 (**B-Ru^{2+}** versus F^-) binding model using the K_A values reported in Table 1 of the main paper.

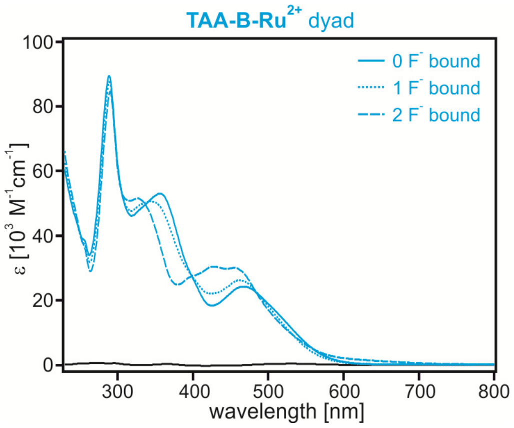

Figure S3. Component spectra used for the fits to the titration data in Figure S3. The individual spectra represent those of **TAA-B-Ru^{2+}** without fluoride (solid line), with one bound fluoride anion (dotted line), and with two bound fluoride anions (dashed line). Inclusion of an additional spectrum (black) caused by TBAF addition to the CH_2Cl_2 solution was necessary for the fits.

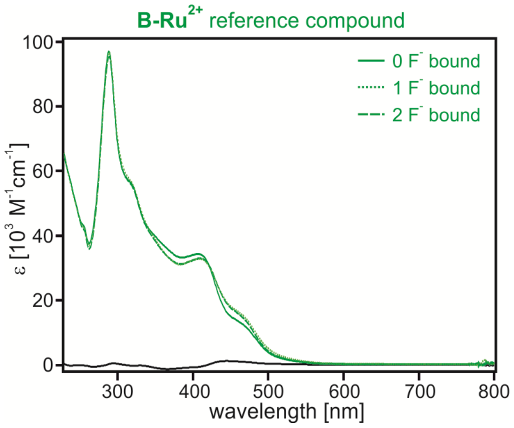

Figure S4. Component spectra used for the fits to the titration data in Figure S4. The individual spectra represent those of **B-Ru^{2+}** without fluoride (solid line), with one bound fluoride anion (dotted line), and with two bound fluoride anions (dashed line). Inclusion of an additional spectrum (black) caused by TBAF addition to the CH_2Cl_2 solution was necessary for the fits.

^{19}F and ^{11}B NMR spectra

Addition of TBAF to CD_2Cl_2 solutions of **TAA-B-Ru^{2+}** and **B-Ru^{2+}** leads to the appearance of a resonance at -170 ppm in the ^{19}F NMR spectrum (Figure S5/S6), and when excess TBAF is present an additional resonance appears at -128 ppm. The former (-170 ppm) is characteristic for dimesitylboron-bound fluoride, the latter (-128 ppm) is due to free F^-.[11a, 11b] Our ^{19}F NMR experiment is unable to distinguish between the two chemically slightly distinct fluoride binding sites present in both **TAA-B-Ru^{2+}** and **B-Ru^{2+}**, but this is not uncommon.[11a, 11b] With ^{11}B NMR spectroscopy one observes the appearance of a resonance at 5 ppm and the disappearance of a resonance at 80 ppm upon fluoride addition (Figure S7/S8), both indicative of F^- binding to dimesitylboron.[11a, 11b, 11e]

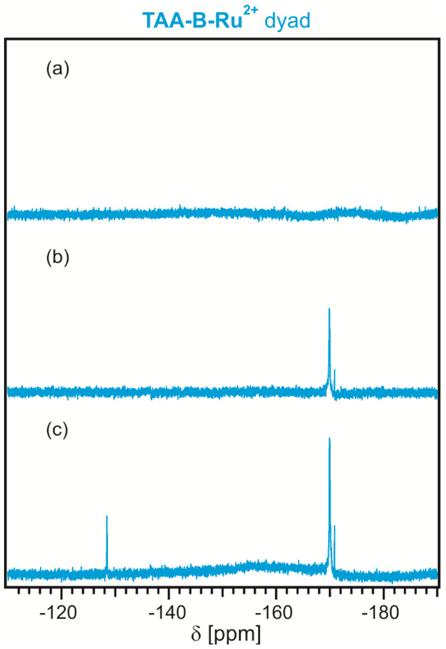

Figure S5. ^{19}F NMR spectra of **TAA-B-Ru^{2+}** in CD_2Cl_2 with various amounts of added TBAF: (a) 0 equivalents of F^-; (b) 1 equivalent of F^-; (c) 4 equivalents of F^-.

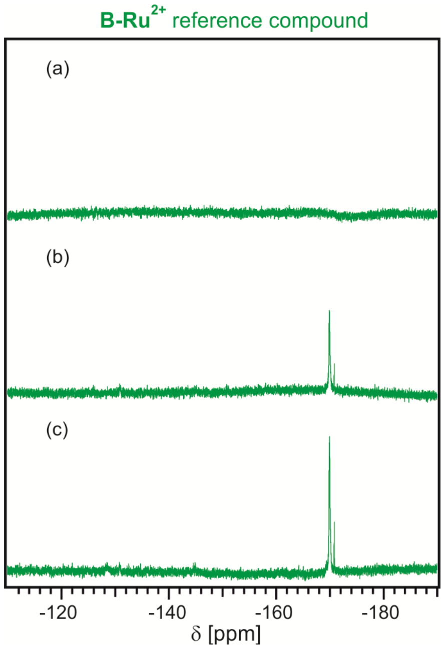

Figure S6. ^{19}F NMR spectra of **B-Ru^{2+}** in CD_2Cl_2 with various amounts of added TBAF: (a) 0 equivalents of F^-; (b) 1 equivalent of F^-; (c) 2 equivalents of F^-.

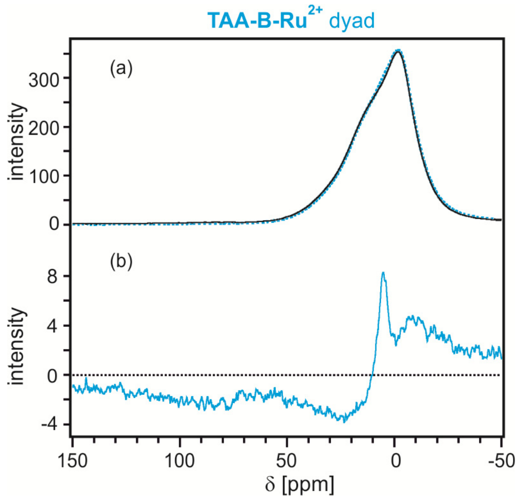

Figure S7. (a) ^{11}B NMR spectra of **TAA-B-Ru^{2+}** in CD_2Cl_2 in absence of TBAF (solid black line) and in presence of 4 equivalents of TBAF (dashed blue line). (b) Difference spectrum between the black and blue lines from (a) in order to subtract the large background signal caused by boron present in the NMR glass tube. The signal from the sample itself is very weak because only a small amount of sample was available for this experiment.

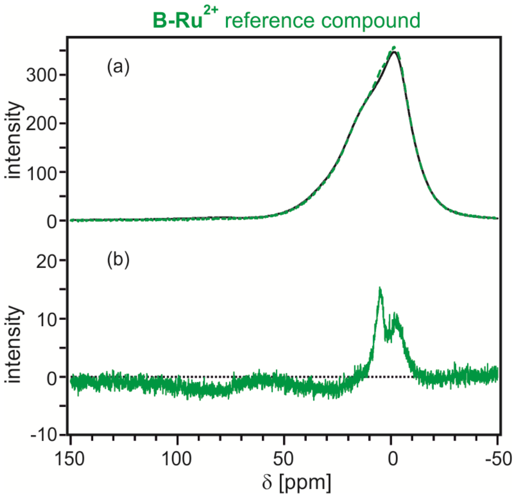

Figure S8. (a) ^{11}B NMR spectra of **B-Ru^{2+}** in CD_2Cl_2 in absence of TBAF (solid black line) and in presence of 4 equivalents of TBAF (dashed green line). (b) Difference spectrum between the black and green lines from (a) in order to subtract the large background signal caused by boron present in the NMR glass tube. The signal from the sample itself is very weak because only a small amount of sample was available for this experiment.

Luminescence of TAA-B-Ru^{2+}, B-Ru^{2+}, and Ru(bpy)$_3^{2+}$ in CH_2Cl_2 in absence of fluoride

The luminescence spectrum of **B-Ru^{2+}** in de-oxygenated CH_2Cl_2 recorded after excitation at 470 nm (green trace in Figure S9a) is similar to the emission spectrum of Ru(bpy)$_3^{2+}$ measured under identical conditions (red trace in Figure S9a), but there is a red-shift of ~30 nm (~1300 cm^{-1}) and an increase in intensity by about 20% in **B-Ru^{2+}** relative to Ru(bpy)$_3^{2+}$ (when comparing solutions which have the same optical density at the excitation wavelength of 470 nm). The bathochromic shift is likely to have its origin in the fact that the lowest MLCT state of **B-Ru^{2+}** involves the bpy ligand with the alkynyl-linked organoboron unit (ligand **22**). The fact that the bathochromic shift between Ru(bpy)$_3^{2+}$ and **B-Ru^{2+}** occurs in combination with a 20% *in*crease of the luminescence intensity indicates that the energy gap law is not applicable to the comparison of Ru(bpy)$_3^{2+}$ and **B-Ru^{2+}**, perhaps because the

emissive excited states are indeed significantly different in these two compounds (MLCT states involving un-substituted bpy (in $Ru(bpy)_3^{2+}$) versus ligand **22** (in **B-Ru^{2+}**)).

What is more, the 20% increase of the luminescence intensity between $Ru(bpy)_3^{2+}$ and **B-Ru^{2+}** is accompanied by a factor of ~3 increase in luminescence lifetime (650 vs. 2040 ns, Table 1), suggesting that the rate constant for radiative excited-state relaxation is substantially higher in $Ru(bpy)_3^{2+}$ than in **B-Ru^{2+}**. Radiative relaxation rate constants are proportional to oscillator strengths hence one might expect to see stronger ^{3}MLCT absorption in $Ru(bpy)_3^{2+}$ than in **B-Ru^{2+}**. However, this is difficult to verify experimentally because the relevant ^{3}MLCT absorption is masked by the ^{1}MLCT absorption tail in both compounds.

The most important piece of information in Figure S9 is the following: The emission of **TAA-B-Ru^{2+}** is completely quenched (Figure S9a), the luminescence decay at 620 nm is instrumentally limited (Figure S9b).

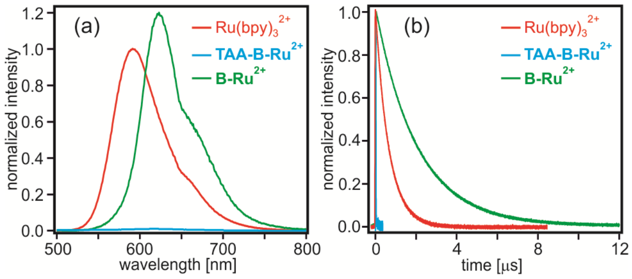

Figure S9. (a) Normalized luminescence spectra obtained after excitation of **TAA-B-Ru^{2+}** (blue trace), **B-Ru^{2+}** (green trace), and $Ru(bpy)_3^{2+}$ (red trace) in de-oxygenated CH_2Cl_2 at 470 nm. The relative intensities of the spectra were corrected for differences in absorbance at the excitation wavelength. (b) Decays of the luminescence intensities at 620 nm in the three samples from (a) measured after excitation at 532 nm with laser pulses of ~10 ns duration.

Transients for TAA-BF$_2^-$-Ru^{2+} and BF$_2^-$-Ru^{2+} in CH_2Cl_2

When four equivalents of fluoride anions are present, the **TAA-BF$_2^-$-Ru^{2+}** species is formed out of **TAA-B-Ru^{2+}** in CH_2Cl_2 (see main paper). There is no evidence that the **TAA-BF$_2^-$-Ru^{2+}** species would undergo photoinduced electron transfer prior to the formation of the long-lived photoproducts detected in Figure 5a of the main paper (solid blue trace). The triarylamine radical cation (TAA^+) has an extinction coefficient at 740 nm which is more than twice as large as the extinction coefficient of the long-lived photoproduct observed in Figure 5a of the main paper (solid blue trace). Consequently, if TAA^+ were indeed formed as a short-lived intermediate, this species would not escape detection in the relevant transient of Figure S10a (blue trace) but it would manifest with an initial decay component exhibiting instrumentally limited kinetics.[12] Since this is not the case and because the same photoproducts are accessible directly after excitation of the **BF$_2^-$-Ru^{2+}** reference molecule under identical conditions, we conclude that in the **TAA-B-Ru^{2+}** dyad, too, these photoproducts are formed directly after excitation at 532 nm. Moreover, and perhaps most importantly, luminescence with a lifetime of 380 ns is observed in presence of 4 equivalents of TBAF (Figure 6 of the main paper). These combined experimental observations indicate that when 2 fluoride anions are bound to **TAA-B-Ru^{2+}**, the resulting **TAA-BF$_2^-$-Ru^{2+}** species no longer exhibits photoinduced electron transfer.

(In presence of 4 equivalents of TBAF, we observed an instrumentally limited decay in addition to the luminescence decay shown in Figure 6b; the same instrumentally limited decay was observed from a reference solution containing only TBAF but no **TAA-B-Ru^{2+}**. Consequently, this instrumentally limited decay was attributed to a fluorescent impurity which is present in the TBAF solution).

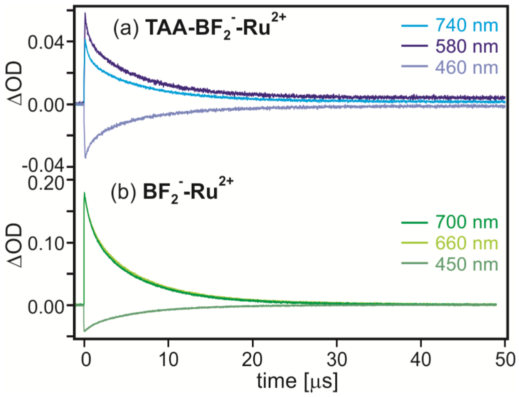

Figure S10. Decays of transient absorption signals measured on $2{\cdot}10^{-5}$ M solutions of (a) **TAA-B-Ru^{2+}** and (b) **B-Ru^{2+}** in de-oxygenated CH_2Cl_2 at 22 °C in presence of 4 equivalents of TBAF. Under these conditions the **TAA-BF$_2^-$-Ru^{2+}** (a) and **BF$_2^-$-Ru^{2+}** (b) species are formed due to tight anion binding (Table 1, see main paper). Excitation occurred at 532 nm with laser pulses of ~10 ns duration. Detection wavelengths were as indicated in the insets.

Additional optical spectroscopic data for B-Ru^{2+} and Ru(bpy)$_3^{2+}$ in absence and presence of F$^-$

The solid red trace in Figure S11b (lower half) is the transient difference spectrum of ^{3}MLCT-excited Ru(bpy)$_3^{2+}$ in de-oxygenated CH_2Cl_2 at 22 °C. The spectrum was recorded by averaging over a time interval of 200 ns immediately after excitation at 532 nm with laser pulses of ~10 ns duration. One observes the common ^{1}MLCT bleach around 450 nm and a band at 370 nm caused by reduced bpy.[13] The transient difference spectrum obtained for **B-Ru^{2+}** under identical conditions (solid green trace in Figure S11a) is rather different. A bleach at 420 nm is detected, but instead of the bpy$^-$ band at 370 nm an intense absorption at 495 nm and two somewhat weaker bands with maxima at 590 and 695 nm appear. The transient absorption signals at 420, 600, and 700 nm all decay with time constants which are within experimental accuracy the same (2040 ns) as the luminescence lifetime detected at 620 nm (Figure S12), hence all of these wavelengths probe the same excited state.

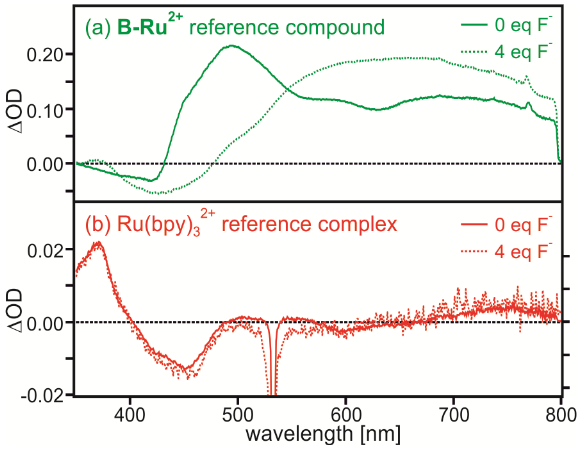

Figure S11. (a) Transient difference spectra measured after excitation of a $2{\cdot}10^{-5}$ M solution of **B-Ru^{2+}** in de-oxygenated CH_2Cl_2 at 532 nm with laser pulses of ~10 ns duration. The spectra were obtained by time-integration over the first 200 ns following the laser pulses. The solid trace was measured in absence of TBAF, the dotted trace was recorded in presence of 4 equivalents of TBAF, leading to the formation of **TAA-BF$_2^-$-Ru^{2+}**. (b) Transient difference spectra obtained for $Ru(bpy)_3^{2+}$ under identical conditions. $Ru(bpy)_3^{2+}$ cannot bind any F^- hence no spectral changes are observed upon TBAF addition.

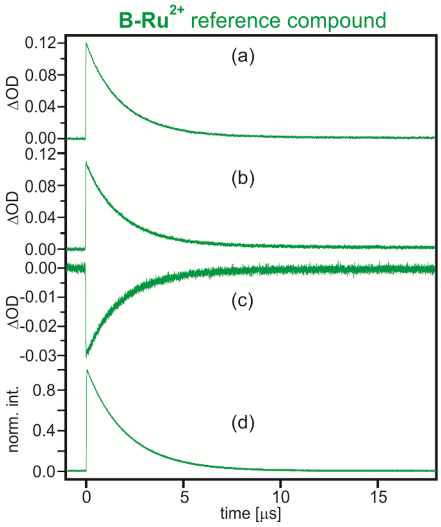

Figure S12. Decays of the transient absorption signals of $2{\cdot}10^{-5}$ M **B-Ru^{2+}** in de-oxygenated CH_2Cl_2 (without TBAF) detected at (a) 700 nm, (b) 600 nm, and (c) 420 nm. (d) Decay of the luminescence intensity detected at 620 nm. In each case, excitation occurred at 532 nm with laser pulses of ~10 ns duration. Analogous decays observed in presence of TBAF are shown in Figure S10b and in Figure S14.

Thus, the lowest excited state of **B-Ru^{2+}** exhibits some of the key characteristics of the emissive ^{3}MLCT state of Ru(bpy)$_3^{2+}$-type complexes (unstructured luminescence, lifetime on the order of ~1000 ns) yet its excited-state absorption spectrum differs from what is detected for Ru(bpy)$_3^{2+}$. We attribute this to the extended π-conjugation of ligand **22** and the involvement of this particular ligand in the relevant MLCT transition.

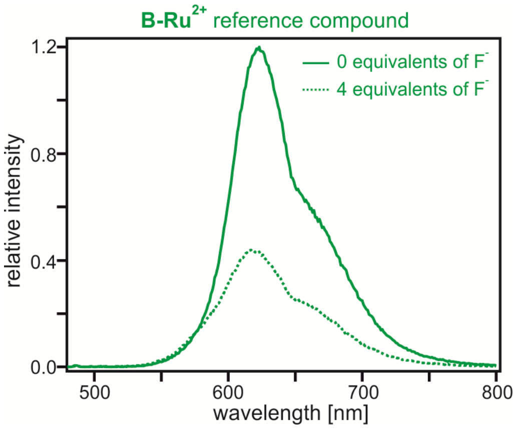

Figure S13. Luminescence spectra obtained after excitation of **B-Ru^{2+}** in de-oxygenated CH_2Cl_2 at 470 nm in absence of TBAF (solid line) and in presence of 4 equivalents of F^- (dotted line; luminescence due to the **BF_2-Ru^{2+}** species). The relative intensities of the spectra were corrected for differences in absorbance at the excitation wavelength.

Based on the association constants from Table 1 of the main paper, addition of 4 equivalents of TBAF to a 2·10^{-5} M solution of **B-Ru^{2+}** in de-oxygenated CH_2Cl_2 leads to a situation in which the vast majority of **B-Ru^{2+}** molecules have two fluoride anions bound, resulting in the **BF_2^--Ru^{2+}** species. When adding 8·10^{-5} M TBAF, the luminescence intensity decreases by approximately a factor of 3 (Figure S13), and the luminescence lifetime in de-oxygenated CH_2Cl_2 shortens from 2040 to 1120 ns (Figure S14d, Table 1). Thus, the decrease in luminescence intensity is slightly more pronounced than the decrease in luminescence lifetime (factor of ~3 versus factor of ~2), suggesting that the rate constants for radiative excited-state relaxation differ by roughly a factor of 1.5 between **B-Ru^{2+}** and **BF_2^--Ru^{2+}**. This in turn leads to the expectation of a factor of ~1.5 difference in oscillator strength in

absorption, but this is difficult to verify experimentally because the relevant ^{3}MLCT absorption is largely masked by the ^{1}MLCT absorption tail in both compounds.

The transient difference spectra obtained for $\mathbf{BF_2^- \text{-} Ru^{2+}}$ and for $Ru(bpy)_3^{2+}$ with 4 equivalents of F^- in de-oxygenated CH_2Cl_2 are shown in Figure S11a/b (dotted traces). While the spectrum of $Ru(bpy)_3^{2+}$ is essentially unaffected by the addition of 4 equivalents of F^- (because $Ru(bpy)_3^{2+}$ cannot bind any F^-), for $\mathbf{BF_2^- \text{-} Ru^{2+}}$ a bleach around 430 nm becomes broader and more prominent than before F^- addition, the band at 495 nm has disappeared and a broad band between 500 and 800 nm has become the dominant feature. The decays of the transient absorption signals at 430, 660, and 700 nm are now biexponential (Figure S14a-c; same data as in Figure S10 but with one detection wavelength being different (430 nm instead of 450 nm)). The faster decay component exhibits within experimental accuracy the same lifetime (1120 ns) as the luminescence detected at 620 nm under the same conditions (Figure S14d). The slower decay component has an average lifetime (τ) of 7160 ns and accounts for ~60% of the total decay at all three detection wavelengths (Figure S14a-c).

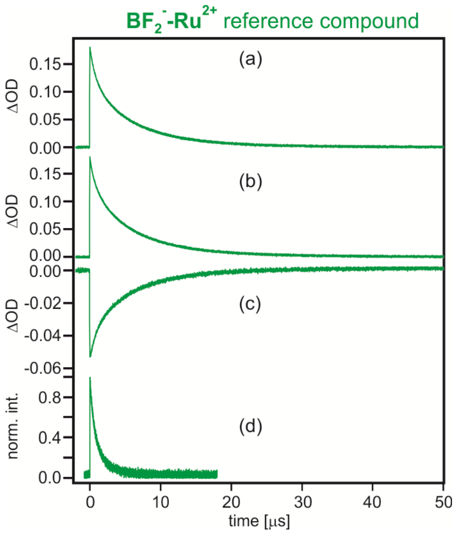

Figure S14. Decays of the transient absorption signals of $2 \cdot 10^{-5}$ M $\mathbf{B\text{-}Ru^{2+}}$ in de-oxygenated CH_2Cl_2 with 4 equivalents of TBAF detected at (a) 700 nm, (b) 660 nm, and (c) 430 nm. (d) ($\mathbf{BF_2^- \text{-} Ru^{2+}}$ is the majority species under these conditions). Decay of the luminescence intensity detected at 620 nm. In each case, excitation occurred at 532 nm with laser pulses of ~10 ns duration.

The two decay components observed in transient absorption must originate from photoexcitation of the **BF_2^--Ru^{2+}** species because the proportion of **B-Ru^{2+}** molecules with only one F^- or no F^- is negligible under the conditions used here (Table 1). Redox chemistry between the organoboron unit and photoexcited $Ru(bpy)_3^{2+}$ is ruled out on the basis of unfavorable electrochemical potentials; the organoboron unit with 2 bound fluoride anions is far too difficult to reduce or to oxidize.[11a, 11c, 14]

The experimental data is compatible with an emissive ^{3}MLCT state (τ = 1120 ns) and a dark state (τ = 7160 ns) which is populated by a nonradiative process. Given its long lifetime and the lack of possible photoredox chemistry, the dark state is most likely a ^{3}IL (IL = intraligand) state (located on ligand **22**) which is populated either via triplet-triplet energy transfer from the ^{3}MLCT state, [15] or via a ^{1}IL state which is fed from the initially excited ^{1}MLCT state (Scheme S2).[16] Given the rapid population of the dark state (< 10 ns), the latter pathway seems more plausible. It is easily conceivable that F^- binding affects the ^{1}IL and ^{3}IL energies such that they become similar to those of the ^{1}MLCT and ^{3}MLCT energies. Photoinduced loss of F^- cannot explain the observed spectral features, and given the high binding constant for F^- (Table 1), the mixture of emissive and dark states (~40% vs. ~60% in proportion) is unlikely to result from co-excitation of **B-Ru^{2+}** with only one bound F^-. There is no indication that ion pairing (between the $Ru(bpy)_3^{2+}$ unit of **B-Ru^{2+}** and excess F^-) would lead to the observed dark state.

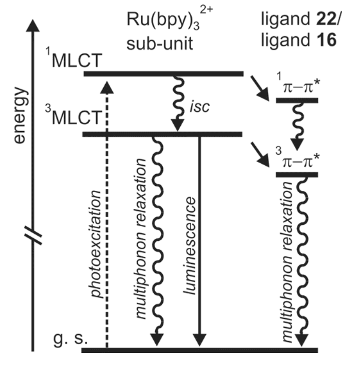

Scheme S2. Energy level diagram illustrating the possible photophysics in **B-Ru^{2+}** and **TAA-B-Ru^{2+}** with 2 bound fluoride anions (**BF_2^--Ru^{2+}** and **TAA-BF_2^--Ru^{2+}**).

Since the ratio between fast and slow decay components observed for **BF_2^--Ru^{2+}** and **TAA-BF_2^--Ru^{2+}** in CH_2Cl_2 stays relatively constant at 40%:60% over a broad wavelength change, the transient absorption spectrum exhibits little changes as a function of delay time.

Cyclic voltammetry in presence of fluoride

The electrochemistry of **TAA-B-Ru^{2+}** in CH_2Cl_2 is less clean when fluoride is present (this seems to be a general problem in the literature), yet our data indicates that the electrochemical potentials for triarylamine oxidation and for ruthenium (bpy) reduction are little affected by F^- binding to the organoboron bridge. Only very few studies have reported electrochemical data for organoboron compounds with bound fluoride anions, presumably because this is generally a difficult experiment to perform.[11a] A related study has reported a cathodic shift of 0.24 V for a Ru(III)/Ru(II) redox couple upon fluoride addition, but in this case the F^- binding site was directly on a cyclometalating ligand.[11c] In **TAA-B-Ru^{2+}** the peripheral boron atoms are relatively far away from the triarylamine and the $Ru(bpy)_3^{2+}$ sub-units, and this can explain why the electrochemical potentials of these two components are rather insensitive to F^- addition.[4]

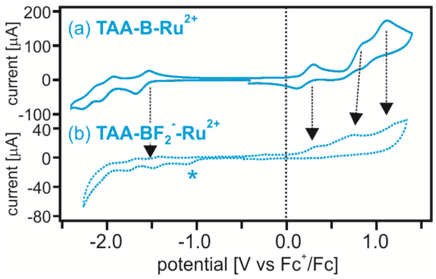

Figure S15. Cyclic voltammograms of (a) **TAA-B-Ru^{2+}** in pure CH_2Cl_2 and (b) **TAA-B-Ru^{2+}** in CH_2Cl_2 with 4 equivalents of TBAF (leading to the formation of **TAA-BF_2^--Ru^{2+}** as a majority species). The potential scan rate was 0.1 V/s in both cases. The wave at -1.1 V vs. Fc^+/Fc in (b) (marked by an asterisk) only appears after an initial oxidative sweep.

References

[1] I. Noviandri, K. N. Brown, D. S. Fleming, P. T. Gulyas, P. A. Lay, A. F. Masters, L. Phillips, *J. Phys. Chem. B* **1999**, *103*, 6713-6722.

[2] H. Shen, K. P. C. Vollhardt, *Synlett* **2012**, 208-214.

[3] a) A. K. Flatt, B. Chen, J. M. Tour, *J. Am. Chem. Soc.* **2005**, *127*, 8918-8919; b) H. H. Jian, J. M. Tour, *J. Org. Chem.* **2005**, *70*, 3396-3424.

[4] C. H. Zhao, A. Wakamiya, Y. Inukai, S. Yamaguchi, *J. Am. Chem. Soc.* **2006**, *128*, 15934-15935.

[5] a) S. Kobayashi, S. Wakumoto, Y. Yamaguchi, T. Wakamiya, K. Sugimoto, Y. Matsubara, Z. Yoshida, *Tetrahedron Lett.* **2003**, *44*, 1807-1810; b) A. Orita, D. An, T. Nakano, J. Yaruva, N. C. Ma, J. Otera, *Chem.-Eur. J.* **2002**, *8*, 2005-2010; c) G. V. Karpov, V. V. Popik, *J. Am. Chem. Soc.* **2007**, *129*, 3792-3793.

[6] U. Ziener, A. Godt, *J. Org. Chem.* **1997**, *62*, 6137-6143.

[7] C. Teng, X. C. Yang, C. Yang, S. F. Li, M. Cheng, A. Hagfeldt, L. C. Sun, *J. Phys. Chem. C* **2010**, *114*, 9101-9110.

[8] M. E. Walther, O. S. Wenger, *ChemPhysChem* **2009**, *10*, 1203-1206.

[9] H. Hart, K. Harada, C. J. F. Du, *J. Org. Chem.* **1985**, *50*, 3104-3110.

[10] J. Hankache, O. S. Wenger, *Chem. Commun.* **2011**, *47*, 10145-10147.

[11] a) C. R. Wade, A. E. J. Broomsgrove, S. Aldridge, F. P. Gabbaï, *Chem. Rev.* **2010**, *110*, 3958-3984; b) H. C. Schmidt, L. G. Reuter, J. Hamacek, O. S. Wenger, *J. Org. Chem.* **2011**, *76*, 9081-9085; c) C. R. Wade, F. P. Gabbaï, *Inorg. Chem.* **2010**, *49*, 714-720; d) Y. Sun, Z. M. Hudson, Y. L. Rao, S. N. Wang, *Inorg. Chem.* **2011**, *50*, 3373-3378; e) Y. M. You, S. Y. Park, *Adv. Mater.* **2008**, *20*, 3820-3826; f) G. Zhou, M. Baumgarten, K. Müllen, *J. Am. Chem. Soc.* **2008**, *130*, 12477-12484; g) A. Sundararaman, K. Venkatasubbaiah, M. Victor, L. N. Zakharov, A. L. Rheingold, F. Jäkle, *J. Am. Chem. Soc.* **2006**, *128*, 16554-16565; h) Y. Kim, F. P. Gabbaï, *J. Am. Chem. Soc.* **2009**, *131*, 3363-3369.

[12] Unless electron transfer products disappear more rapidly than they are formed, but there is no physical basis to assume that this would be the case.

[13] a) A. Yoshimura, M. Z. Hoffman, H. Sun, *J. Photochem. Photobiol. A* **1993**, *70*, 29-33; b) P. Müller, K. Brettel, *Photochem. Photobiol. Sci.* **2012**, *11*, 632-636.

[14] Y. Sun, S. N. Wang, *Inorg. Chem.* **2010**, *49*, 4394-4404.

[15] a) G. J. Wilson, A. Launikonis, W. H. F. Sasse, A. W. H. Mau, *J. Phys. Chem. A* **1997**, *101*, 4860-4866; b) J. A. Simon, S. L. Curry, R. H. Schmehl, T. R. Schatz, P. Piotrowiak, X. Q. Jin, R. P. Thummel, *J. Am. Chem. Soc.* **1997**, *119*, 11012-11022; c) B. Maubert, N. D. McClenaghan, M. T. Indelli, S. Campagna, *J. Phys. Chem. A* **2003**, *107*, 447-455; d) J. Gu, J. Chen, R. H. Schmehl, *J. Am. Chem. Soc.* **2010**, *132*, 7338-7346; e) J. R. Schoonover, D. M.

Dattelbaum, A. Malko, V. I. Klimov, T. J. Meyer, D. J. Styers-Barnett, E. Z. Gannon, J. C. Granger, W. S. Aldridge, J. M. Papanikolas, *J. Phys. Chem. A* **2005**, *109*, 2472-2475; f) S. Boyde, G. F. Strouse, W. E. Jones, T. J. Meyer, *J. Am. Chem. Soc.* **1989**, *111*, 7448-7454; g) A. El-ghayoury, A. Harriman, A. Khatyr, R. Ziessel, *J. Phys. Chem. A* **2000**, *104*, 1512-1523; h) M. B. Majewski, N. R. de Tacconi, F. M. MacDonnell, M. O. Wolf, *Chem.-Eur. J.* **2013**, *19*, 8331-8341.

[16] a) R. T. F. Jukes, V. Adamo, F. Hartl, P. Belser, L. De Cola, *Coord. Chem. Rev.* **2005**, *249*, 1327-1335; b) B. He, O. S. Wenger, *Inorg. Chem.* **2012**, *51*, 4335-4342.
